# Role of Second Quinone Binding Site in Proton Pumping by Respiratory Complex I

**DOI:** 10.3389/fchem.2019.00221

**Published:** 2019-04-09

**Authors:** Outi Haapanen, Amina Djurabekova, Vivek Sharma

**Affiliations:** ^1^Department of Physics, University of Helsinki, Helsinki, Finland; ^2^Institute of Biotechnology, University of Helsinki, Helsinki, Finland

**Keywords:** redox chemistry, proton transport, electron transport, density functional calculations, molecular dynamics, cell respiration

## Abstract

Respiratory complex I performs the reduction of quinone (Q) to quinol (QH_2_) and pumps protons across the membrane. Structural data on complex I have provided spectacular insights into the electron and proton transfer paths, as well as into the long (~30 Å) and unique substrate binding channel. However, due to missing structural information on Q binding modes, it remains unclear how Q reduction drives long range (~20 nm) redox-coupled proton pumping in complex I. Here we applied multiscale computational approaches to study the dynamics and redox chemistry of Q and QH_2_. Based on tens of microseconds of atomistic molecular dynamics (MD) simulations of bacterial and mitochondrial complex I, we find that the dynamics of Q is remarkably rapid and it diffuses from the N2 binding site to another stable site near the entrance of the Q channel in microseconds. Analysis of simulation trajectories also reveal the presence of yet another Q binding site 25–30 Å from the N2 center, which is in remarkable agreement with the electron density observed in recent cryo electron microscopy structure of complex I from *Yarrowia lipolytica*. Quantum chemical computations on the two Q binding sites closer to the entrance of the Q tunnel reveal redox-coupled protonation reactions that may be important in driving the proton pump of complex I.

## Introduction

**Figure 1:**
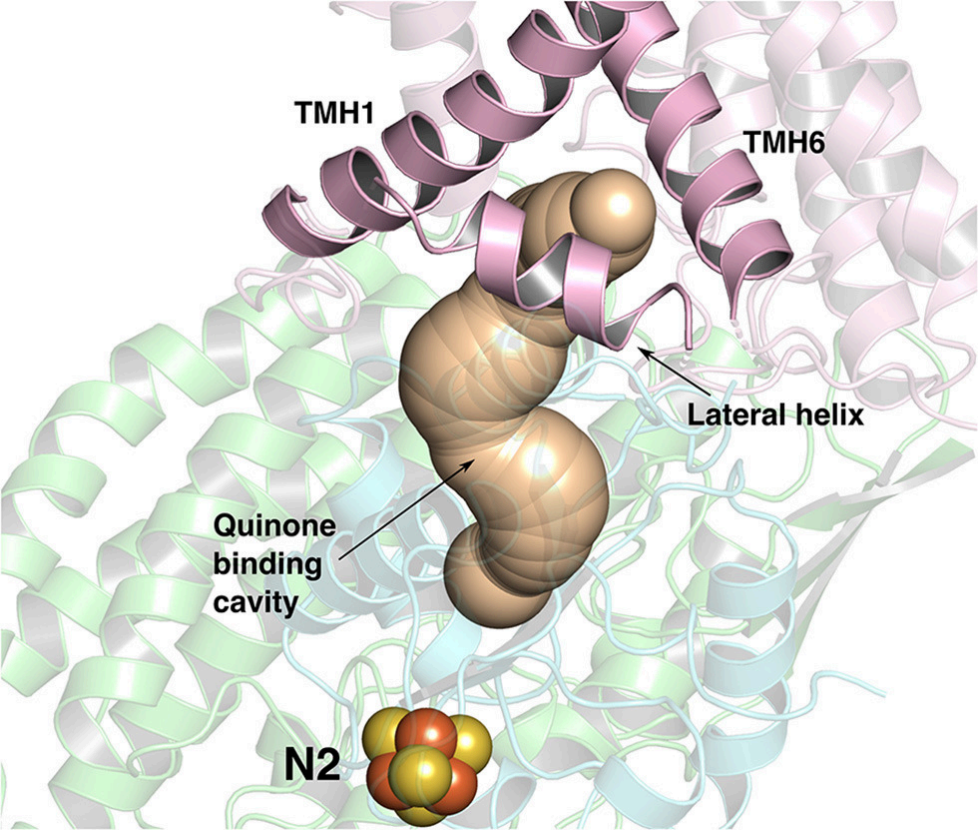
The ~35 Å long Q-binding tunnel of respiratory complex I from *Thermus thermophilus* (PDB 4HEA) in which a Q molecule can bind within electron transfer distance to the terminal FeS cluster, N2. Subunits Nqo8, Nqo6, and Nqo4 that form the Q binding site are displayed in pink, blue and green ribbons, respectively. The three helices (transmembrane helices/TMHs 1 and 6, and lateral helix between TMHs 1 and 2) of Nqo8 subunit form the Q channel opening. CAVER (Pymol) (Chovancova et al., 2012) was used to make the figure.

The first enzyme in the electron transport chains of mitochondria and many bacteria is respiratory complex I, which transfers electrons released upon NADH oxidation to a quinone (Q) molecule, reduction of which to quinol (QH_2_) drives the proton pumping across the membrane (Supplementary Figure 1 and Figure 1) (Wikstrom et al., 2015). Interestingly, the reactions catalyzed by complex I are reversible (Vinogradov, 1998), but how the coupling between electrons and protons is achieved, remains unknown. It is especially intriguing because data from atomic resolution structures (Baradaran et al., 2013; Zickermann et al., 2015; Fiedorczuk et al., 2016; Gu et al., 2016; Zhu et al., 2016; Guo et al., 2017; Agip et al., 2018; Blaza et al., 2018) show that the pumping occurs as far as ~200 Å from the “active site” of redox reactions, a notion also supported by a number of site-directed mutagenesis studies (Euro et al., 2008; Nakamaru-Ogiso et al., 2010; Michel et al., 2011). Despite rather extensive biochemical, biophysical and structural data on complex I that led to a number of mechanistic proposals (Sazanov, 2015; Wikstrom et al., 2015; Hirst and Roessler, 2016; Wirth et al., 2016), the basic principles of redox-coupled proton pumping are still hotly debated. Computational approaches have been pivotal in providing molecular details of possible mechanistic coupling between redox reactions and proton pumping in complex I (Haapanen and Sharma, 2018; Kaila, 2018), and also in other electron transfer complexes that use Q or its analogs as substrates (Saito et al., 2013; Barragan et al., 2015, 2016). Recently, by employing free energy calculations on the structure of complex I from *Thermus thermophilus* (Warnau et al., 2018), we identified four Q binding sites in the Q tunnel (1, 1', 2, and 2'). The sites 1 and 1' are similar to the Q binding sites proposed based on the structural work by Sazanov and Brandt groups, respectively (Baradaran et al., 2013; Zickermann et al., 2015). The site 1 is the location at which quinone is converted to quinol by electron transfer from N2, which is the terminal FeS center in the long chain of FeS clusters in complex I (Figure 1; Sazanov and Hinchliffe, 2006). The quinol formed at site 1, is found to diffuse rapidly to site 1' based on earlier modeling and simulation approaches (Sharma et al., 2015; Haapanen and Sharma, 2017; Warnau et al., 2018). The site 1' has also been implicated in the two Q-binding-site mechanism proposed by Brandt and Zickermann (Zickermann et al., 2015). The function of two other sites 2 and 2', which are ca. 25 and 35 Å away from the critical Tyr87 (*Thermus* enzyme numbering), and which are seen as distinct energy minima in free energy calculations, remain unknown.

It is likely that the reduction of Q to quinol (QH_2_) at the site near N2 occurs by simultaneous abstraction of protons from the surrounding residues (His38/Asp139 and Tyr87 of Nqo4 subunit). Redox potential measurements (Verkhovsky et al., 2012; Verkhovskaya and Wikstrom, 2014) as well as multiscale calculations (Sharma et al., 2015; Warnau et al., 2018) reveal that the site has low redox potential (< −300 mV). Therefore, no energy is released upon electron transfer from NADH to Q bound at the N2 site, which is required to pump protons across the membrane (Wikstrom et al., 2015). Instead, it has been suggested that the reactions that occur beyond Q reduction at the N2 site drive proton pumping. The question then arises what are those reactions? Do they involve *pure* conformational transitions or if long-ranged electrostatics and/or proton transfer reactions are also at play? Such molecular aspects remain entirely unknown. Building upon our earlier proposals (Wikstrom et al., 2015; Haapanen and Sharma, 2018), we have here performed extensive molecular dynamics (MD) simulations on small model systems of bacterial and mitochondrial complex I to study the microsecond dynamics of Q/QH_2_. Quantum chemical calculations performed on the lesser known Q binding sites in the Q chamber provide novel and testable viewpoints on the proton pumping mechanism of complex I.

## Materials and Methods

We performed long time scale fully atomistic MD simulations of bacterial and mammalian complex I structures from *Thermus thermophilus* [*T.t*., PDB id 4HEA (Baradaran et al., 2013)] and *Bos taurus* [*B.t*., PDB id 5LC5(Zhu et al., 2016)], respectively. To achieve long time scale dynamics of Q and QH_2_ in the ~35 Å long Q-binding cavity, we restricted the size of the model systems by choosing only the core subunits that surround the Q tunnel. The chosen subunits are Nqo7/ND3, Nqo8/ND1, Nqo4/49kDa, Nqo5/30kDa, Nqo6/PSST, Nqo9/TYKY for *T.t*./*B.t*., and Nqo16 (*T.t*. only). The embedding of protein in POPC lipid bilayer was achieved with CHARMM-GUI (Jo et al., 2007, 2008, 2009; Brooks et al., 2009; Wu et al., 2014; Lee et al., 2016) tools by using the OPM aligned structures (Lomize et al., 2012). We modeled the quinone/quinol molecules (Q_1_/Q_10_ see Supplementary Table 1) into the system near reduced N2 cluster (Haapanen and Sharma, 2017). After our recurrent observation of the additional Q sites along the Q cavity, we used these sites as the starting point for several other simulations (see Supplementary Table 1). We solvated the entire membrane-protein-Q system with TIP3 (MacKerell et al., 1998) water molecules and with 0.1 M NaCl salt concentration. All amino acids were kept in their standard protonation states, except His38 of Nqo4 subunit, which was protonated in oxidized Q simulations of *T.t*. models [see ref. (Sharma et al., 2015)]. In the case of QH_2_ simulations, His38 was modeled neutral and Tyr87 (Nqo4) was kept anionic based on our earlier work (Sharma et al., 2015). Due to system truncation, the membrane facing Glu74/68 of subunit Nqo7/ND3 were also protonated to avoid artificial hydration at the membrane-protein interface. The modeled Q/QH_2_ was relaxed by a short minimization keeping constraints (5–50 kcal mol^−1^ Å^−2^) on the protein heavy atoms. Following this, 100 ps NVT and 1 ns NPT equilibration runs were performed while keeping the constraints on the protein (with membrane and solvent free). After these relaxation protocols, we removed the constraints, minimized the system and performed 100 ps NVT and 10 ns NPT runs to equilibrate the temperature and pressure of the system. The CHARMM force field parameters for ubiquinone (Galassi and Arantes, 2015), reduced FeS clusters present in Nqo6/PSST and Nqo9/TYKY subunits (Chang and Kim, 2009), protein, water and lipids (MacKerell et al., 1998; Mackerell et al., 2004; Klauda et al., 2010) were used, and MD simulations were performed by using the GROMACS (Abraham et al., 2015) software at 310 K and 1 atm temperature and pressure, respectively. In the production runs, we used Nose-Hoover thermostat (Nosé, 1984; Hoover, 1985) and Parrinnello-Rahman barostat (Parrinello and Rahman, 1980, 1981). The PME method (Darden et al., 1993), as implemented in GROMACS, was used to treat the electrostatic interactions. The model systems consist of ~230,000 atoms and the time step of the simulations was 2 fs, achieved by using the LINCS algorithm (Hess et al., 1997) as implemented in GROMACS. Total simulation time is ~70 μs, which corresponds to ca. 10 million CPU hours, and all simulations are listed in Supplementary Table 1. Analysis of root mean square deviation plots show that the simulation systems are stabilized and system truncation does not cause destabilization (Supplementary Figure 2). The contact analysis was performed to identify amino acid residues that interact with the Q head group at various Q binding locations. For the purpose, we chose amino acid sidechains that were within 5 Å of the Q head group ring.

Snapshots from classical MD simulations were selected that showed water-protein based connectivity between the Q head group at site #5 and the N side of the membrane (see below). These snapshots were then used to perform the Quantum Mechanical/Molecular Mechanical Molecular Dynamics (QM/MM MD) simulations, using additive QM/MM coupling with electrostatic embedding, to elucidate the redox-coupled protonation changes that may take place at the site next to the entrance of the Q tunnel. Prior to QM/MM MD simulations, a 500–1,000 steps classical energy minimization was performed followed by a 100 step QM/MM energy minimization for all setups. The simulations were performed using QCHEM (Shao et al., 2015)/CHARMM (Brooks et al., 2009) packages, using the density functional theory (DFT) with B3LYP functional (Becke, 1988, 1993; Lee et al., 1988) and 6–31G^*^ basis set (Supplementary Table 2) by considering the dispersion corrections (Grimme et al., 2010) with 1 fs time step and at 310 K. Larger values of time step resulted in system instability due to energy fluctuations, and were not used. In *B.t*. QM/MM simulations, the QM regions consisted of residues – Ser44 and Arg77 from PSST, and Asp51, Lys54, Glu204 and Asn212 from ND1 subunit (setups B1-B3 in Supplementary Table 2). In *T.t*. simulations, the QM region comprised Arg36, Asp62, Lys65, Ser66 and Lys69 from Nqo8, and Asp59 from Nqo6 (setups T1-T3 in Supplementary Table 2). In both setups, Q head group and surrounding water molecules were also considered in the reactive QM region. Link atoms were introduced between CA-CB of protein residues, and between C11 and C12 of the Q molecule. The protonation dynamics was observed as a consequence of the reduction of QM region by one and two electrons (Supplementary Table 2), thereby leading to the formation of semiquinone and quinol, respectively.

We also performed QM/MM MD simulations on site #4 where a QH_2_ molecule was modeled (see also Supplementary Table 1). Oxidation of QH_2_ at this site may be triggered by an oxidized Q molecule at site #5 as proposed recently in ref. (Haapanen and Sharma, 2018). In our earlier work, we modeled a Q molecule at site #4 in several different redox-protonation states, and observed water-protein based connection between the Q tunnel and the first putative proton transfer channel in the antiporter-like subunits (Haapanen and Sharma, 2017). Moreover, the recent cryo EM data shows unassigned electron density that remarkably fits our Q binding site #4 (see below). Overall, these data rationalize modeling and simulation of Q molecules at sites 4 and 5. In the hybrid QM/MM simulations on site #4, the reactive QM region comprised the QH_2_ head group, surrounding water molecules and residues Glu24, Arg25, Arg195, Glu202, and Glu227 from ND1 subunit and Asp70 and Arg71 from PSST subunit. Three different systems were constructed by selecting different QM regions (setups B4-B6 in Supplementary Table 2).

Additional DFT calculations were performed on the selected region of the second Q binding site (site#5, ca. 215 atoms). The geometry optimizations were performed with BP86 (Perdew, 1986; Becke, 1988) functional and def2-SVP (Weigend and Ahlrichs, 2005) basis set by considering dispersion corrections (Grimme et al., 2010). The CB atoms of protein residues were kept fixed. This was followed by single-point and spin density calculations using def2-TZVP basis set (Weigend and Ahlrichs, 2005) and two different density functionals B3LYP (Becke, 1988, 1993; Lee et al., 1988) and TPSSh (Tao et al., 2003). The dispersion corrections were applied during energy minimization as well as single point calculations and COSMO model was used to describe the dielectric constant of 4 during single point calculations (Klamt and Schüürmann, 1993). All calculations were performed by using TURBOMOLE (7.2)^1^.

To identify the p*K*_a_ changes that may occur due to protein/Q dynamics, we also performed p*K*_a_ calculations on MD simulation snapshots using Propka software (Olsson et al., 2011; Sondergaard et al., 2011).

The simulation snapshots from several MD runs are available for download on the link; https://doi.org/10.5281/zenodo.1498266.

## Results and Discussion

### Dynamics of Q and QH_2_ in the Q Tunnel

**Figure 2:**
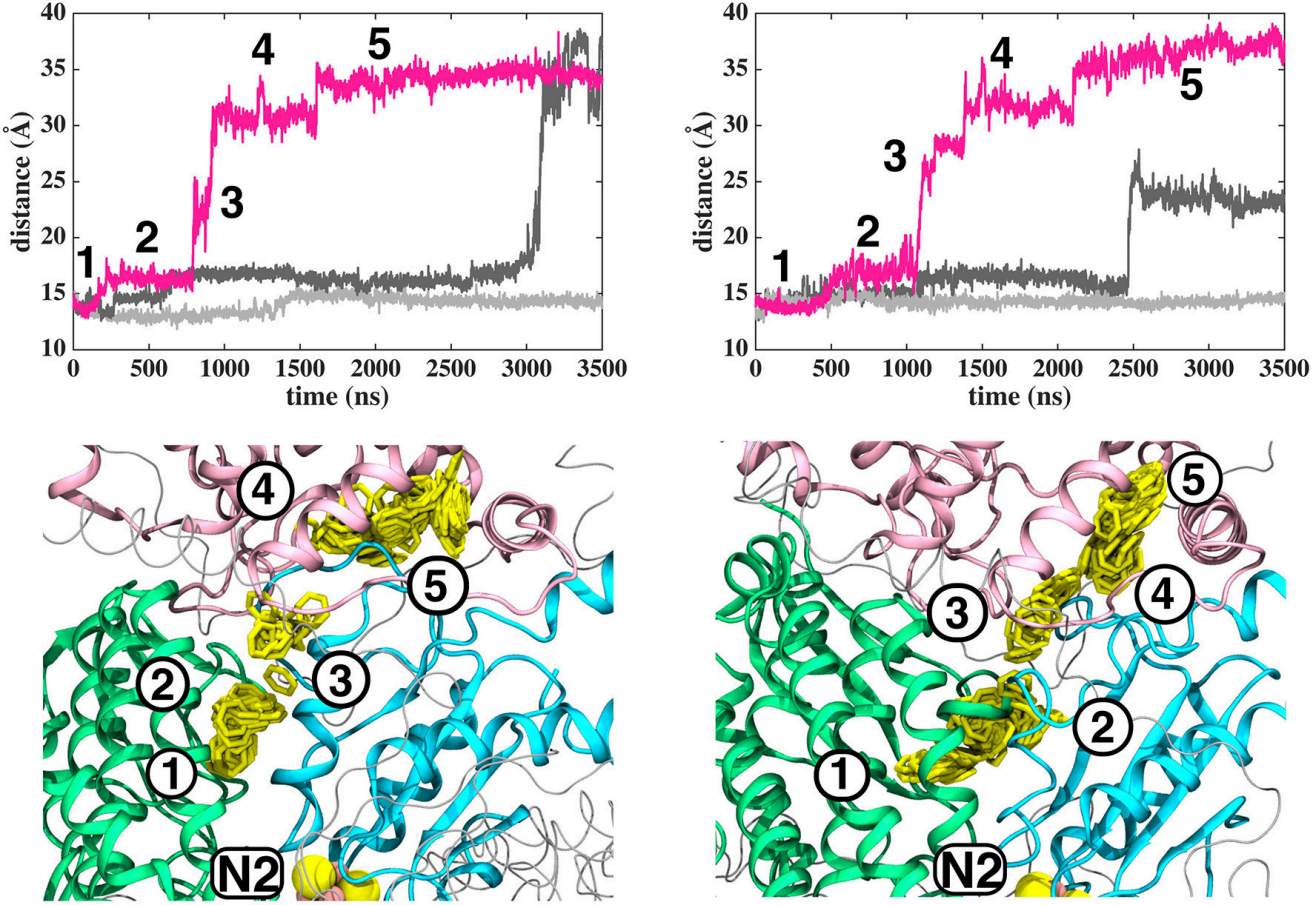
Dynamics of Q (Q_10_) molecule in the channel like cavity of complex I revealed by simulations of *T.t*. and *B.t*. enzymes (left and right panels, respectively). Upper panels show the distance between the center-of-mass of the six carbon atoms of the Q head group ring and the center-of-mass of N2 FeS cluster (Fe and S atoms) from simulation setups I and II with three independent simulation replicas in different colors (Methods). Lower panels display the transient arrests of Q (numbered 1 to 5) in the Q-tunnel, shown by the head group (yellow) position with 10 ns interval.

Based on long time scale MD simulations of mitochondrial (*B.t*.) and bacterial (*T.t*.) complex I, we find that an oxidized Q (Q_10_) molecule diffuses within microseconds from the first stable N2 binding site (#1 in Figure 2) to another stable site located at the entrance of the Q channel, ca. 35 Å away from reduced N2 cluster (#5 in Figure 2). The location of this site is nearly identical to the low energy Q binding site that has been found based on short timescale free energy simulations (Warnau et al., 2018) (For Q binding site nomenclature in this work and earlier Warnau et al., 2018, see Supplementary Figures 3, 11). While the oxidized Q diffuses rapidly in the tunnel, analogous simulations of a QH_2_ molecule show that after exiting the site #1, it interacts strongly with the acidic residues of 49 kD and ND1 subunits, which limits its long timescale dynamics (Supplementary Figures 4, 5). When modeled out of the local trap, QH_2_ also moves rapidly closer to the site #4 (Supplementary Figures 4, 5), where it maintains stable interactions with the charged residues of ND1 subunit (see below). Interestingly, MD simulations of both long-tailed Q and QH_2_ molecules reveal tight coupling between the dynamics of protein and its rapid diffusion in the Q chamber (Supplementary Videos S1, S2) in which highly conserved histidine carrying β1-β2 loop of subunit Nqo4 (H34/38 and H55/59 in *T.t*. and *B.t*., respectively), as well as the Q-cavity facing loops of Nqo6 and Nqo8 subunits rearrange as Q travels through the channel.

**Figure 3:**
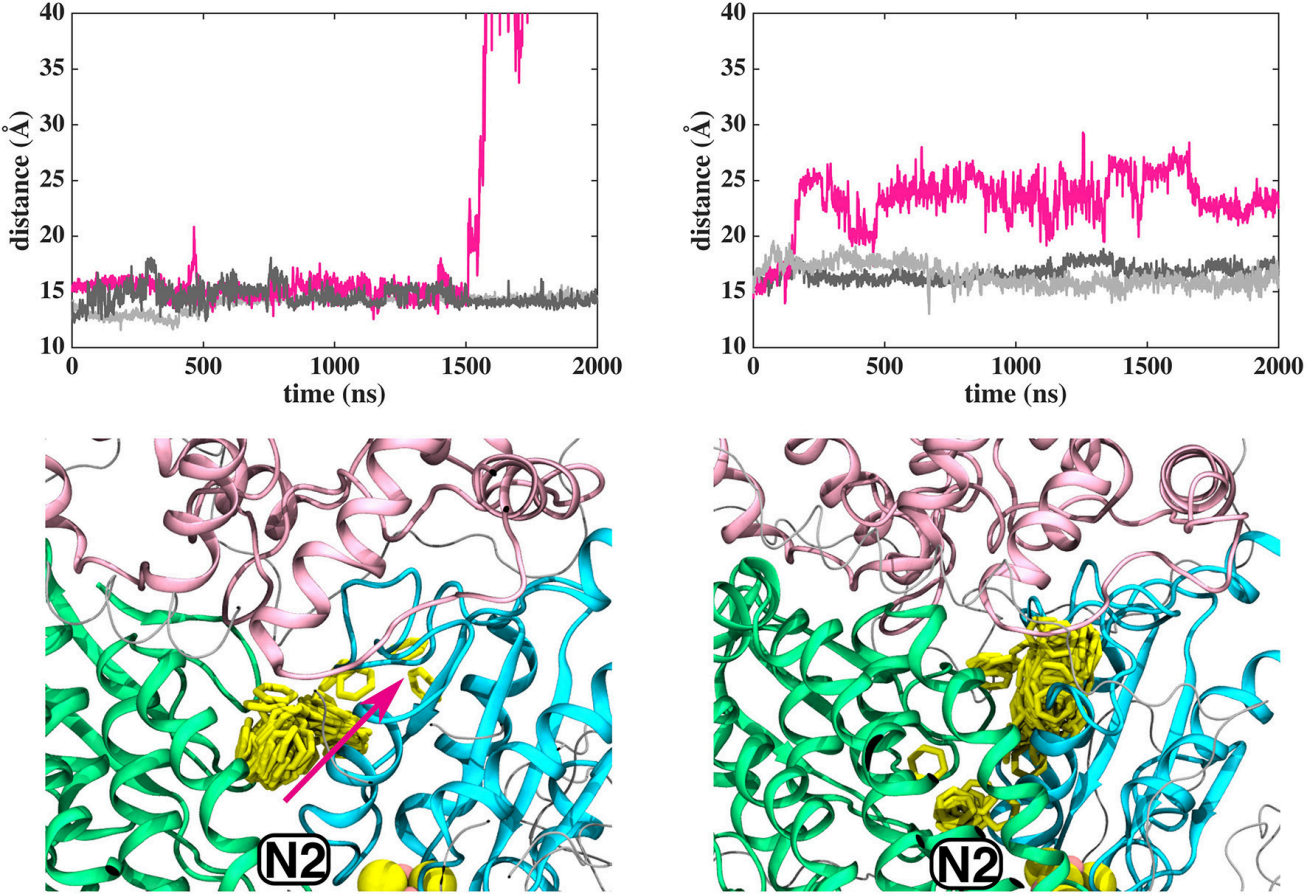
Dynamics of Q_1_ molecule in the Q binding cavity observed in our MD simulations of *T.t*. and *B.t*. enzymes (left and right panels). Upper and lower panels are the same as in Figure 2. The pink arrow shows the region through which Q_1_ exited in *T.t*. simulation (see also magenta trace).

**Figure 4:**
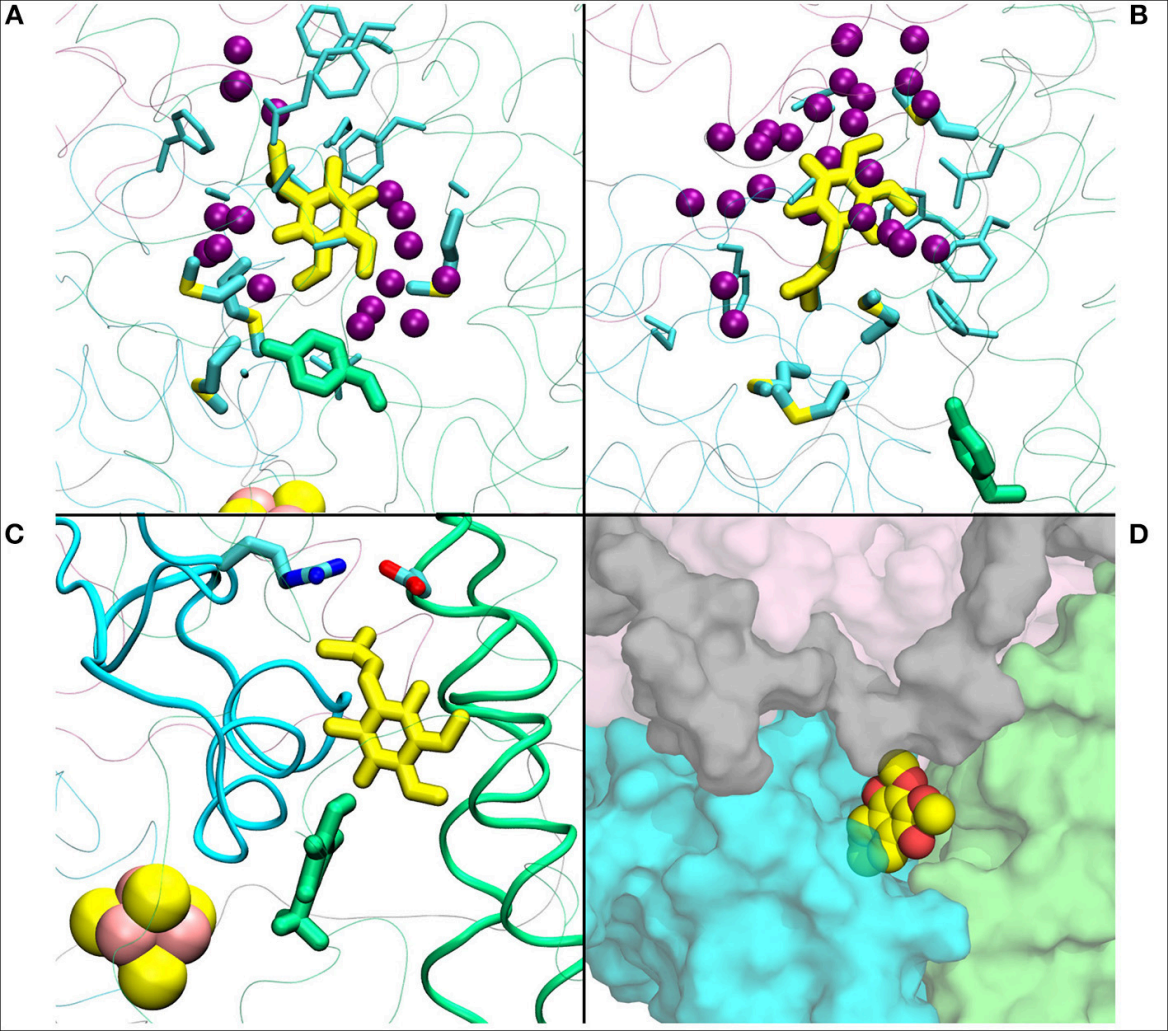
**(A)** Q_1_ molecule in its regular orientation with the headgroup toward Tyr108 (in green licorice, *B.t*.). **(B)** shows the Q_1_ molecule in its “flipped” conformation. Due to the missing long isoprenoid tail, the small Q_1_ molecule has space to flip around in the cavity. **(C)** demonstrates a restriction formed by charged residues from Nqo4 and Nqo6 subunits due to the missing long isoprenoid tail. **(D)** shows the alternative exit path of the Q_1_ molecule through a space between subunits Nqo4 (green), Nqo6 (cyan), Nqo7(gray), and Nqo8 (pink).

In contrast to the diffusion of long-tailed Q molecules through the entire Q chamber, short-tailed quinones (Q_1_) display somewhat different dynamics. Overall, they are seen to bind tightly near the N2 center (site #1, Figure 3). However, we find that a Q_1_ molecule can in fact rotate inside the Q chamber and can even be released to the solvent through alternative exit route formed by subunit-subunit interface (Figures 3, 4). This suggests the presence of cavities in the protein through which Q or its analogs can diffuse in and out of complex I active site, which is in agreement with the data from chemical biology approaches that show substrates can access the Q redox active site near N2 through alternative routes (Uno et al., 2019). Our long timescale simulation data also concur with the proposal from Fedor et al. (2017), who, based on kinetic experiments, suggested that long isoprenoid tail assists in the correct anchoring of the ubiquinone in the Q chamber.

**Figure 5:**
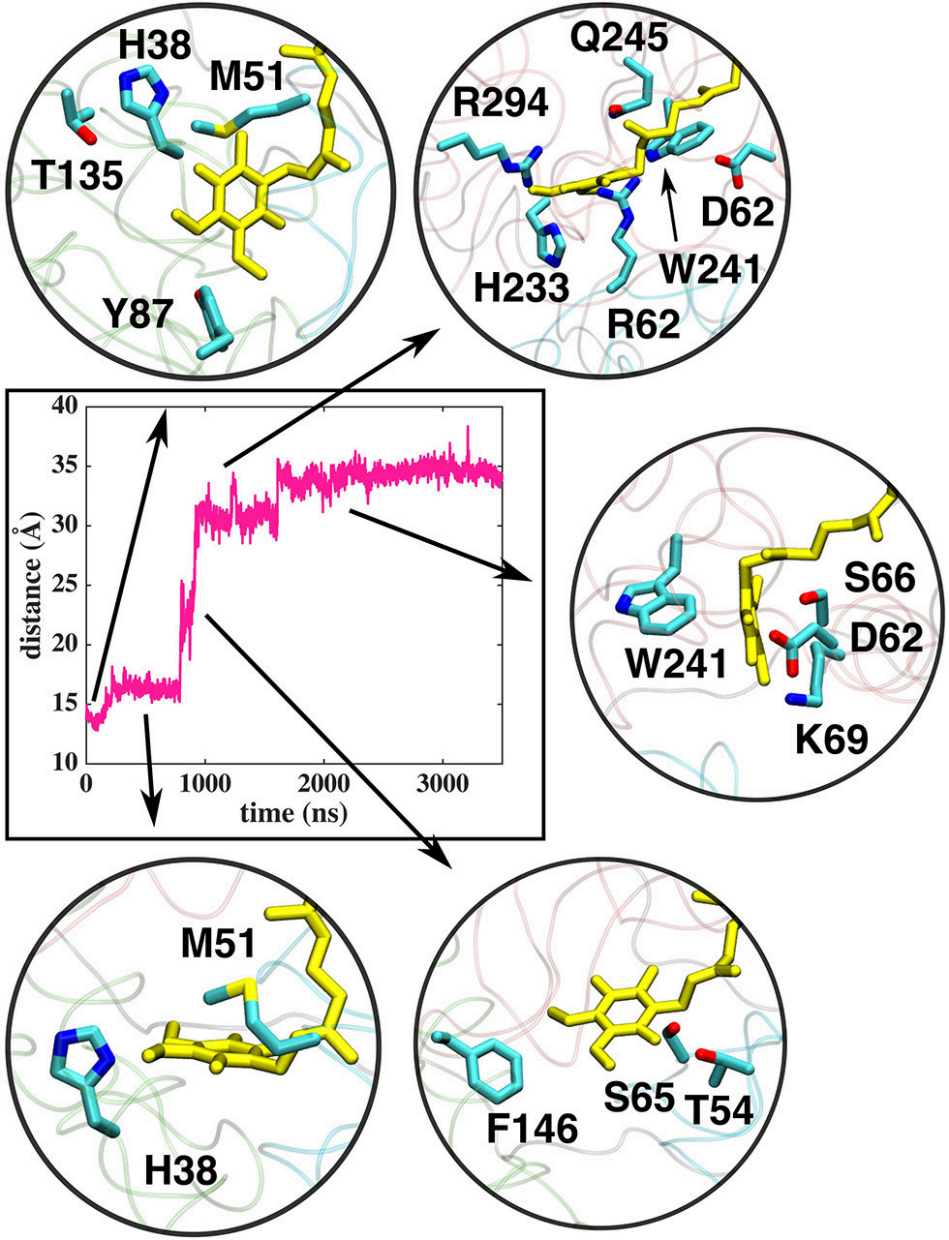
Interactions of the Q head group with the amino acid residues in the Q tunnel. The data shown is based on simulation setup I. Amino acid residues are shown in atomic representation (carbon–cyan, sulfphur–yellow, oxygen–red, and nitrogen–blue) and Q molecule in yellow licorice.

The classical simulations provide a firsthand view of the Q dynamics in ~35 Å long tunnel like cavity of mitochondrial and bacterial complex I, and show that the path taken by long-tailed Q is rather similar in different simulation replicas, albeit the timing of escape differs (Figure 2). Even though the diffusion of Q away from N2 is rapid (~μs), it does not occur without transient halts (sites) along the Q-tunnel (numbered 1–5 in Figure 2). We characterized each of these five Q binding sites and analysis of simulation trajectories revealed conserved amino acid residues that interact with the Q molecule during its diffusion along the Q chamber (see Tables 1, 2, and Figure 5), some of which are potential candidates for site-directed mutagenesis studies.

**Table 1:** Amino acid residues that contact the Q head group at the second Q-binding site (# 5) for at least 30% of simulation time (setups III–VI).

**Subunit**	***T.t*.**	***B.t***
Nqo8 / ND1	F28	V17
	T32	T21
	**E35**	**E24**
	**R36**	**R25**
	P59	P48
	**D62**	**D51**
	**A63**	**A52**
	**K65**	**K54**
	S66	L55
	I239	G128
	W241	F220
	A242	A221
	Q245	F224
	Y249	Y228
Nqo6 / PSST	W37^*^	W46^*^
	Q74^*^	Q82^*^

*Residues in bold refer to those that have been biochemically characterized*.

* *Based on the last ~2 and ~1.5 μs of simulation data from setups III and IV, respectively, in which Q head group switches its position (see also Figure 11 and Supplementary Figure 9)*.

**Table 2:** Amino acid residues that interact with the Q head group during its diffusion in the Q channel.

**Transient halt**	**Subunit**	**Residue**
1st	Nqo4	H38
		V40
		Y87
		T135
	Nqo6	A47
		I48
		M51
2nd	Nqo4	H38
	Nqo6	M51
		V67
3rd	Nqo4	F146
	Nqo6	T54
		S65
4th	Nqo6	R62
	Nqo8	D62
		H233
		W241
		Q245
		R294
5th	Nqo8	D62
		S66
		K69
		W241
		A242

*The data is based on simulation setup I (see also Figure 2 and Supplementary Figure 4). Only those residues are listed whose sidechains were within 5 Å of the Q head group ring for at least 30% of simulation time spent at a transient halt*.

### Q Binding Sites in the Q Tunnel

**Figure 6:**
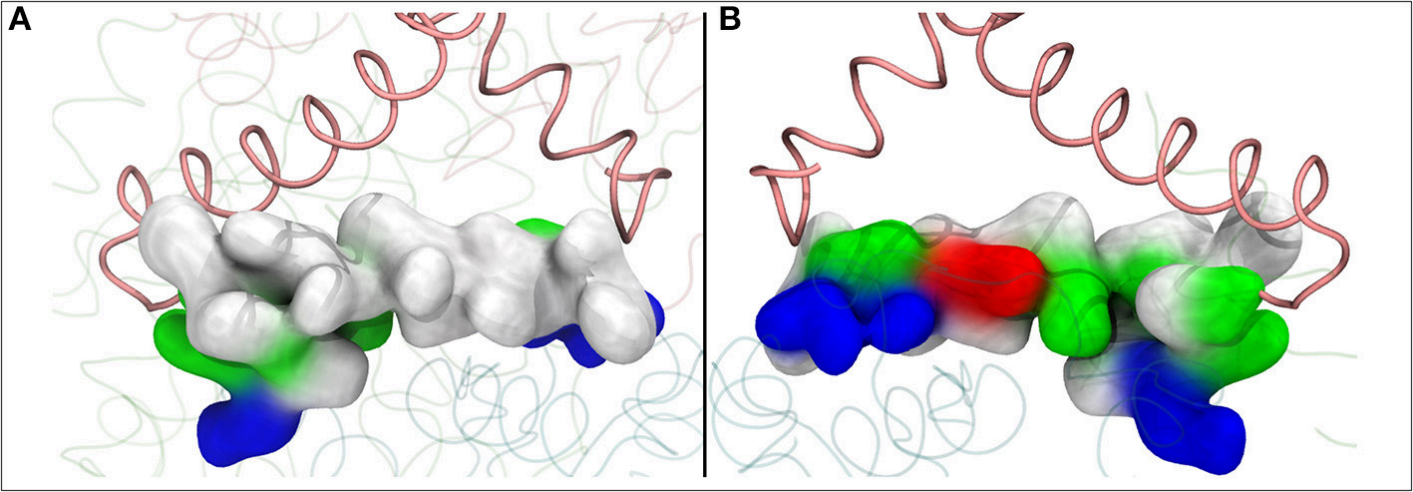
The lateral helix of subunit Nqo8 (*T.t*.), which forms part of the second Q binding site at the entrance of Q-tunnel, is shown in surface representation. View from outside **(A)** and inside **(B)** complex I, parallel to the membrane-bound subunits. **(A)** shows highly hydrophobic surface of the helix facing the lipid bilayer, whereas the side that faces the Q head group at the second Q binding site is highly charged **(B)**.

**Figure 7:**
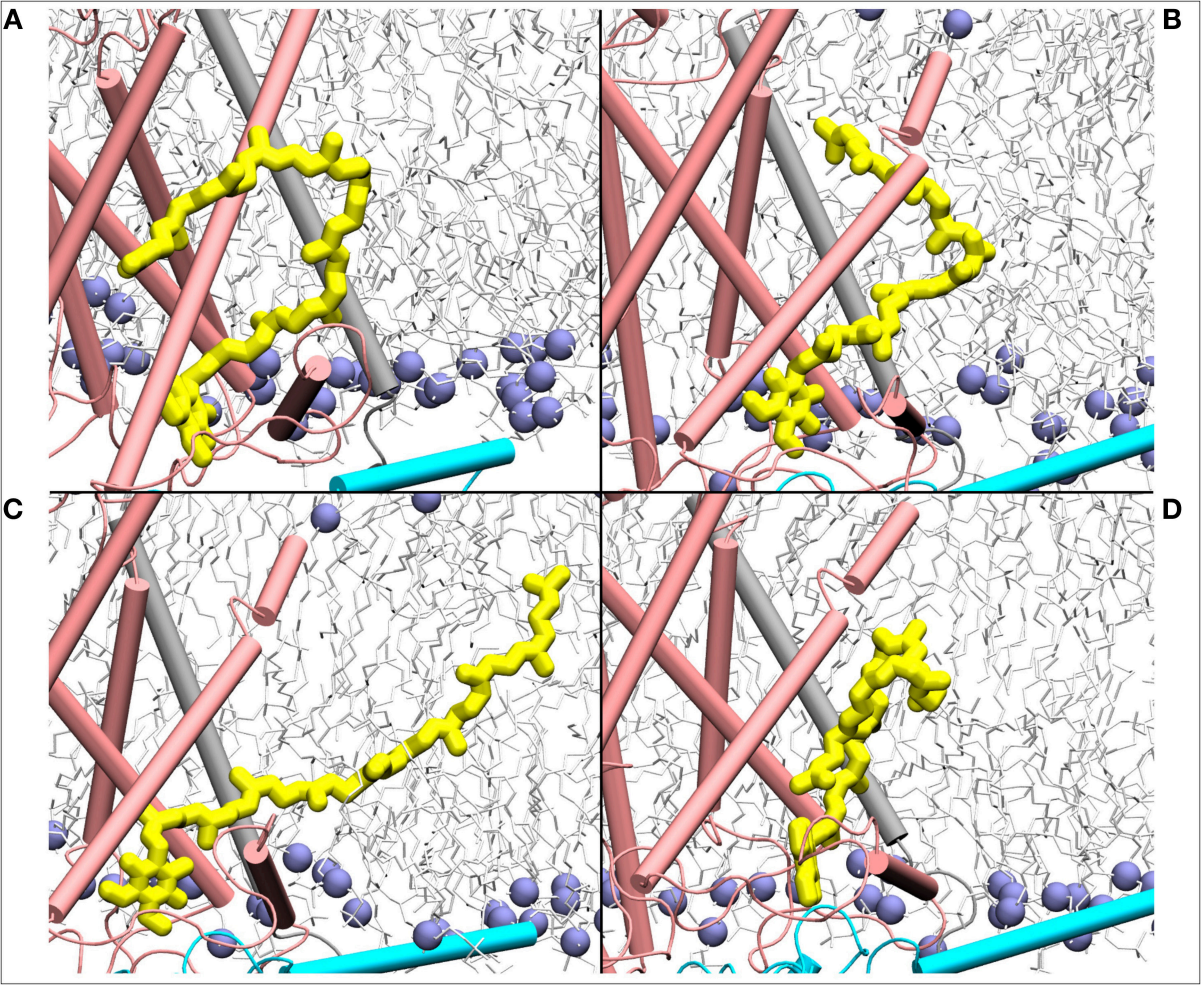
Mixing of ubiquinone (Q_10_, yellow) tail with the lipid molecules shown as simulation snapshots **(A–D)**. The ubiquinone tail, when Q head group binds at the site near entrance of Q tunnel, is highly dynamic and acquires several different poses ranging from completely extended to collapsed. Subunits Nqo6/7/8 are shown in cyan, gray and pink, respectively. Lipid phosphorus atoms are shown as ice blue spheres.

The Q molecule is seen escaping the binding sites 1 and 2 (Figure 2) and ending up at a region next to the Q-tunnel opening (site #5 in Figures 2, 5, see also Supplementary Videos S1, S2). At this location the Q head group interacts with a number of amino acid residues that are conserved in bacterial and mitochondrial enzymes (Table 1) and is stabilized next to the charged surface of the lateral helix of Nqo8 (ND1) subunit (Figures 1, 6). As a result of Q diffusion and associated protein conformational changes, variation in protonation pattern of several key amino acid residues is observed (Supplementary Figure 6). Interestingly, replacement of number of titratable residues located in this region (E35/E24, R36/R25, D62/D51 *T.t*./*B.t*. numbering) is known to affect the structure and function of complex I(Zickermann et al., 1998; Sinha et al., 2009; Patsi et al., 2012), which would strongly support the proposed Q binding site [see also (Warnau et al., 2018)]. Moreover, at this location the Q head group is partly buried in the protein interior and is ca. 10–20 Å from the aqueous phase at the N side of the membrane, whereas its lipid-like tail is solvated in the membrane phase, where it acquires compact and extended conformations (Figure 7). Our microsecond long MD runs show that the Q molecule remains bound to the site (Figure 2 and Supplementary Videos S1, S2), which is in agreement with the potential of mean force data from Umbrella Sampling simulations (Warnau et al., 2018).

**Figure 8:**
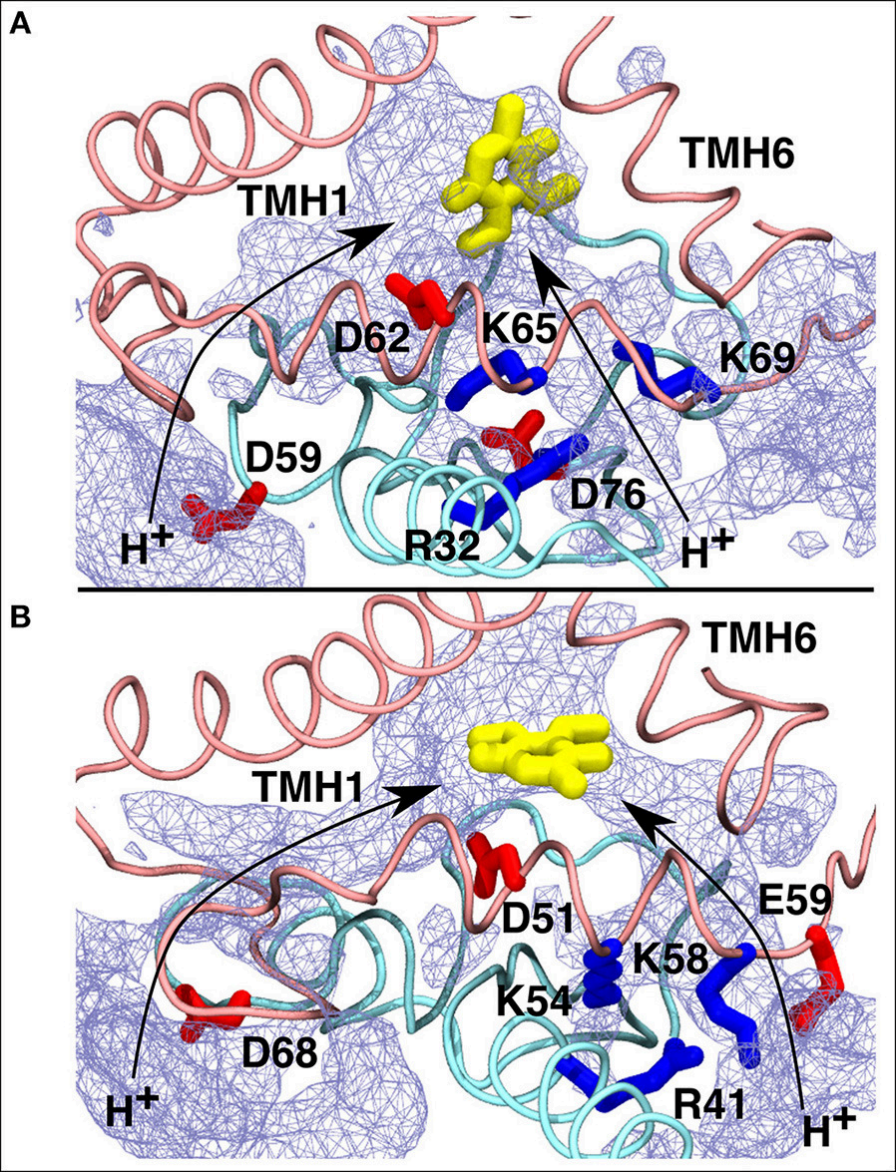
Proposed proton transfer pathways (curved arrows) from the N side of the membrane to the second Q binding site observed in *T.t*. **(A)** and *B.t*. **(B)** simulations. Conserved charged residues from subunits Nqo8/ND1 (pink) and Nqo6/PSST (cyan) that participate in proton channels are shown, together with the Q head group in yellow. Water occupancy (~20%) is displayed as an ice-blue mesh.

To shed further light on site #5, we analyzed the water occupancy in the region near the Q tunnel entrance and found two dominant paths that connect the Q head group with the aqueous phase at the N side of the membrane (Figure 8). The observed pathways, shown as an average water occupancy (Figure 8), comprise several conserved charged amino acid residues from subunits Nqo6/PSST and Nqo8/ND1, some of which are known to be critical for Q-reductase activity (Table 1), thus raising a possibility that Q bound at this site may undergo redox/protonation reactions (Wikstrom et al., 2015; Haapanen and Sharma, 2018).

**Figure 9:**
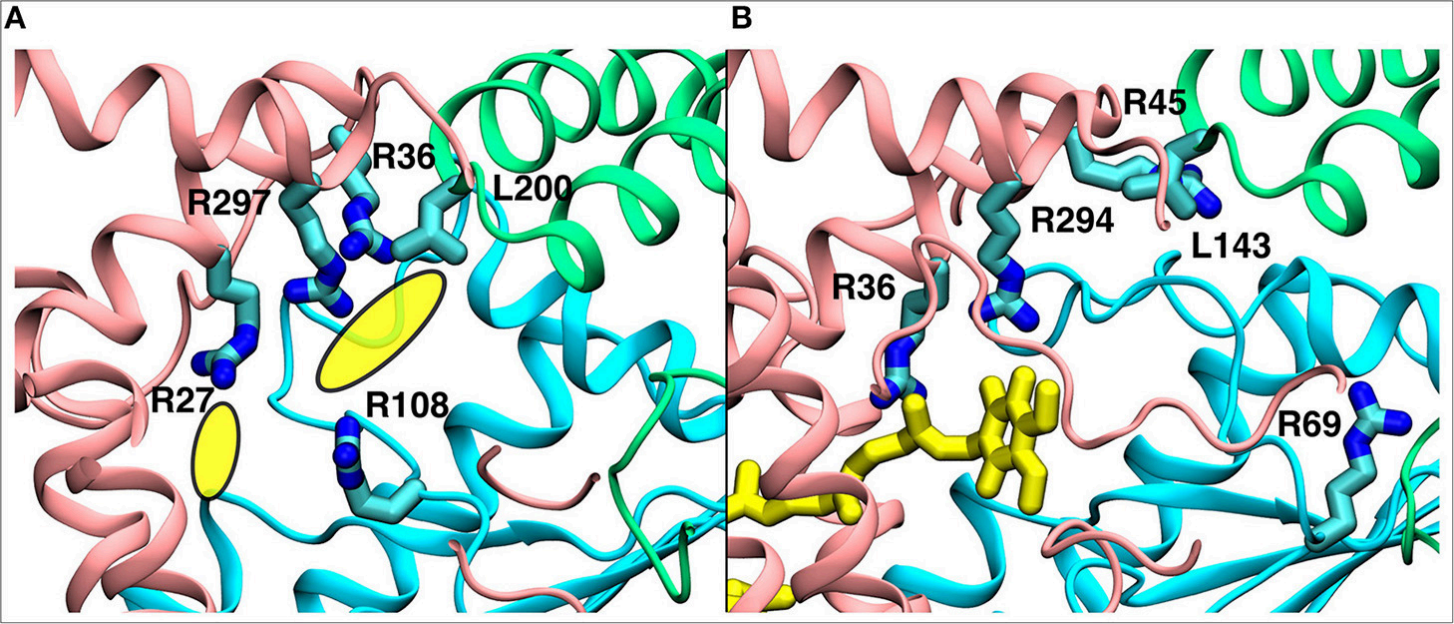
A comparison of the observed electron density in *Yarrowia lipolytica* structure (Parey et al., 2018) (PDB 6GCS, **A**) with the quinone binding site #4 in *Thermus thermophilus* MD simulation **(B)**. The yellow ellipses in the left panel describe the unassigned electron density, whereas the quinone at site #4 is shown in yellow in the right panel. The data indicate functional significance of the site, which is studied by multiscale simulations (see main text). The three-dimensional coordinates of simulation snapshot shown in **(B)** are available for download (see Methods).

We find that the binding site #4 (Figure 2), which is further away from the Q tunnel entrance, remarkably overlaps with the unassigned electron density observed in the recent cryo EM structure of complex I from *Yarrowia lipolytica* (Parey et al., 2018; Figure 9). At this site the Q molecule interacts with a number of positively charged amino acid residues (Figure 9) and is closer to the acidic loop of ND1/Nqo8 subunit. Analysis of hydration profiles in this region reveals connection between the Q head group and charged residues connecting the “E channel” (Baradaran et al., 2013; Supplementary Figure 7). Moreover, p*K*_a_ calculations suggest changes in protonation behavior of residues (Supplementary Figure 6), some of which are known to be critical based on site-directed mutagenesis data and are also “hot-spot” for disease mutations [see references in Baradaran et al. (2013)].

The two Q binding sites (#4 and #5) are found here by studying the long timescale behavior of Q/QH_2_ molecules and are similar to the distinct minima observed in free energy calculations (sites 2 and 2', respectively Warnau et al., 2018. Notably, the two sites are < 14 Å apart (Supplementary Figure 3) and are next to the critical “E-channel” (Baradaran et al., 2013) that connects the Q cavity with the antiporter-subunits. Moreover, given the observation that reactions beyond N2 binding site are responsible for energy coupling, we hypothesize that the redox-coupled proton transfer reactions at these sites are critical in driving the proton pump.

### Redox-Coupled Protonation Reactions at the Sites Closer to the Entrance of Q Tunnel

**Figure 10:**
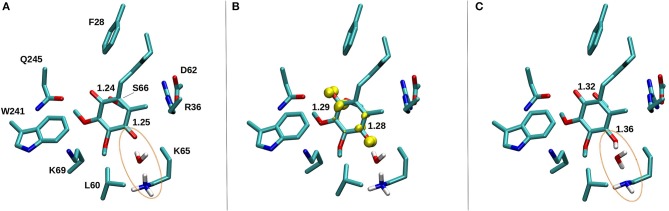
DFT optimized structure of oxidized Q **(A)**, SQ **(B)**, and Q^2−^ **(C)** species. The C = O bond distances of the Q head group are shown for all three species, along with the spin density (yellow) on anionic SQ in **(B)**. Water mediated protonation of Q occurs only upon two-electron reduction **(C)** from the nearby conserved K65 from subunit Nqo8 (also observed in QM/MM MD simulations, see Supplementary Videos S4, S5).

Based on earlier suggestions (Wikstrom et al., 2015; Haapanen and Sharma, 2018), we envisage that the tightly bound oxidized Q at the second binding site (#5 in Figure 2) undergoes reduction from a trapped QH_2_ (at site #4, Figure 2 and Supplementary Figure 4), which may be coupled to proton transfer reactions in the channels identified in this work (Figure 8 and Supplementary Figure 7). In order to study the redox-coupled protonation of Q at the Q binding site #5, we performed hybrid QM/MM MD simulations on snapshots chosen from classical MD simulations of both mitochondrial and bacterial complexes (see Methods). The data from hybrid simulations reveal rapid protonation of unstable doubly reduced quinol (Q^2−^) species from the surrounding residues, forming QH^−^. In contrast, a single electron transfer to an oxidized Q, forming semiquinone (SQ), is not coupled to protonation (Supplementary Videos S3, S4). This scenario is observed in a number of independent simulations performed on the structures of bovine and *Thermus* complexes, thereby suggesting that our conclusions are robust. The data is also supported by independent cluster DFT calculations, which show formation of SQ occurs without proton extraction from the surrounding residues or water molecules (Figure 10). Most importantly, the proton transfer to Q^2−^ occurs from the conserved charged residues that participate in channel formation at the N side of the membrane (Figure 8) and takes place in a stepwise manner also the involving water molecules (Supplementary Video S5). In *T.t*. simulations, two charged residues (Lys65 from Nqo8 and Asp76 from Nqo6) participate in proton relay, the latter of which is connected to the N-side of the membrane (Figure 8). Both residues are critical for enzymatic activity (Zickermann et al., 1998; Garofano et al., 2003), therefore, supporting their role in reduction-coupled proton uptake from the N-side (see also p*K*_a_ data Supplementary Figure 6). Similarly, in *B.t*. simulations, a rapid proton transfer is observed from D51 of ND1 subunit, which has also been found to be critical for the activity (Zickermann et al., 1998; Sinha et al., 2009; Patsi et al., 2012). Overall, the existence of proton pathways, which connect the Q head group at the entrance site with the N-phase of the membrane, and along which explicit proton transfer reactions can occur, support the viewpoint that the observed Q binding site may undergo potential redox-coupled protonation reactions.

To complement the reduction of Q at site #5 from QH_2_ at site #4 (Wikstrom et al., 2015; Haapanen and Sharma, 2018), we also performed additional QM/MM simulations on the latter location (see Methods). The data reveal oxidation-coupled protonation of highly conserved charged amino acids that are near the site #4 (Supplementary Figures 7, 8). We note that this Q binding location is similar to what has been studied earlier based on pure classical simulation approaches (Haapanen and Sharma, 2017) and also remarkably matches the electron density observed in recent cryo EM structure of yeast complex I (Parey et al., 2018). The multiscale calculations performed here provide independent support to the putative Q binding site and to the models of proton pumping discussed in refs. Wikstrom et al. (2015) and Haapanen and Sharma (2018).

Earlier, EPR experiments led to the identification of two semiquinone signals, SQ_Nf_ and SQ_Ns_ (Ohnishi et al., 2012). The SQ_Nf_ signal originates due to spin-spin coupling between the reduced N2 FeS cluster and SQ, whereas SQ_Ns_ signal, which is not sensitive to the membrane potential, was found to be associated with a Q binding site ca. 30 Å from N2. Based on our calculations, this site may correspond to the second stable Q-binding site #5 found in our simulations. Interestingly, EPR experiments suggest the SQ_Ns_ species to be anionic in nature, which is what our QM/MM simulations and cluster DFT calculations also show.

### Dynamics of Q/QH_2_ at the Entrance Site

**Figure 11:**
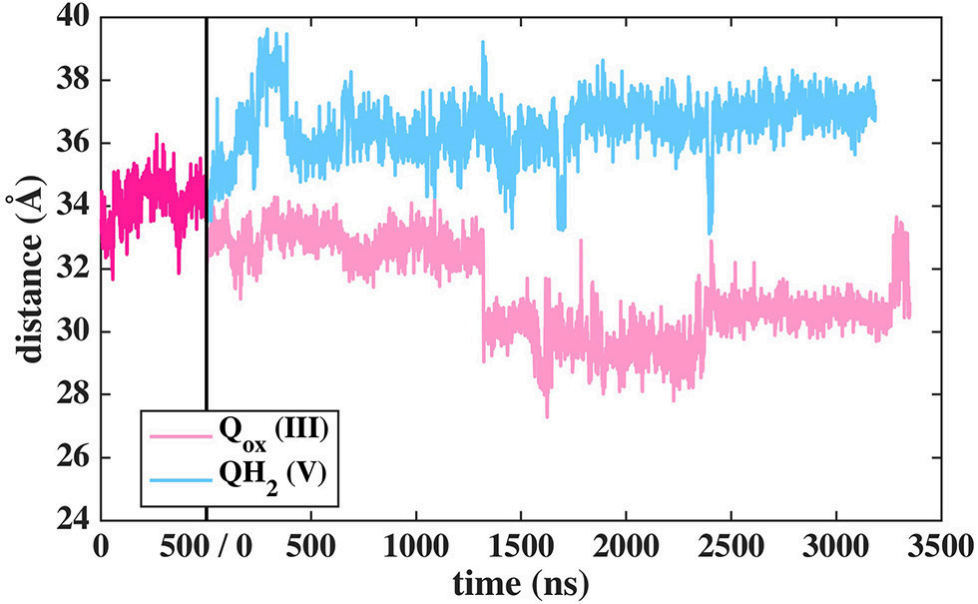
Dynamics of two different Q species at the second binding site in *T.t*. simulation shown as distance between N2 and Q with respect to simulation time. The dark pink plot corresponds to 2,000–2,500 ns of MD data from Q setup I (see Figure 2).

The binding and dynamics of Q/QH_2_ molecules at sites 1 and 4 has been studied earlier by means of classical simulations (Sharma et al., 2015; Haapanen and Sharma, 2017). To understand the binding and dynamics of different Q species at the Q binding site #5, we performed long time scale MD simulations on *T.t*. and *B.t*. setups by modeling different Q species at the latter site. The data reveal that oxidized Q binds tightly at the second Q binding site in comparison to the weaker binding of QH_2_ molecule (seen as the departure from the N2 center, Figure 11 and Supplementary Figure 9). Even though a complete dissociation of QH_2_ molecule is not observed due to limited sampling and energetic barriers (Warnau et al., 2018), the data support the notion that second Q binding site binds oxidized Q more tightly than QH_2_. A simulation of anionic quinol (QH^−^), which forms upon redox-coupled protonation of oxidized Q at the second Q binding site, reveals formation of protein-water mediated routes between the Q head group and the N-side aqueous phase, which may allow protonation of QH^−^ to form QH_2_ at this site (Supplementary Figure 10).

### Two Q Molecules in the Q Tunnel—Is It Possible?

**Figure 12:**
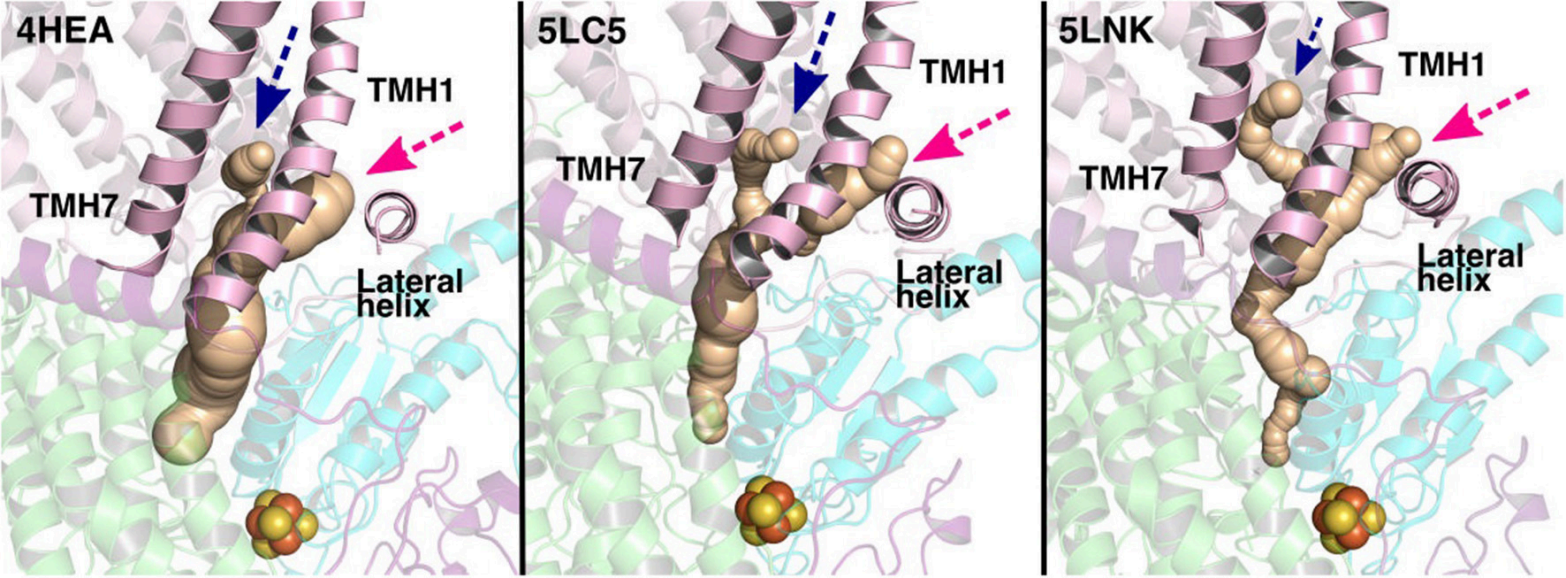
Potential Q tunnels in complex I structures. The widely recognized Q binding channel is marked with pink arrows that opens next to the lateral helix of Nqo8/ND1 subunit. The alternative cavity for the quinone tail between TMH1 and TMH7 in Nqo8/ND1 is shown in three different structures (marked by blue arrows). Our computational results (see also Figure 13) suggest the latter channel as a potential candidate for Q tail dynamics. The figure was prepared with CAVER software using probe radius 0.7 Å and the starting point for cavity computation was H38/59 and Y87/108.

**Figure 13:**
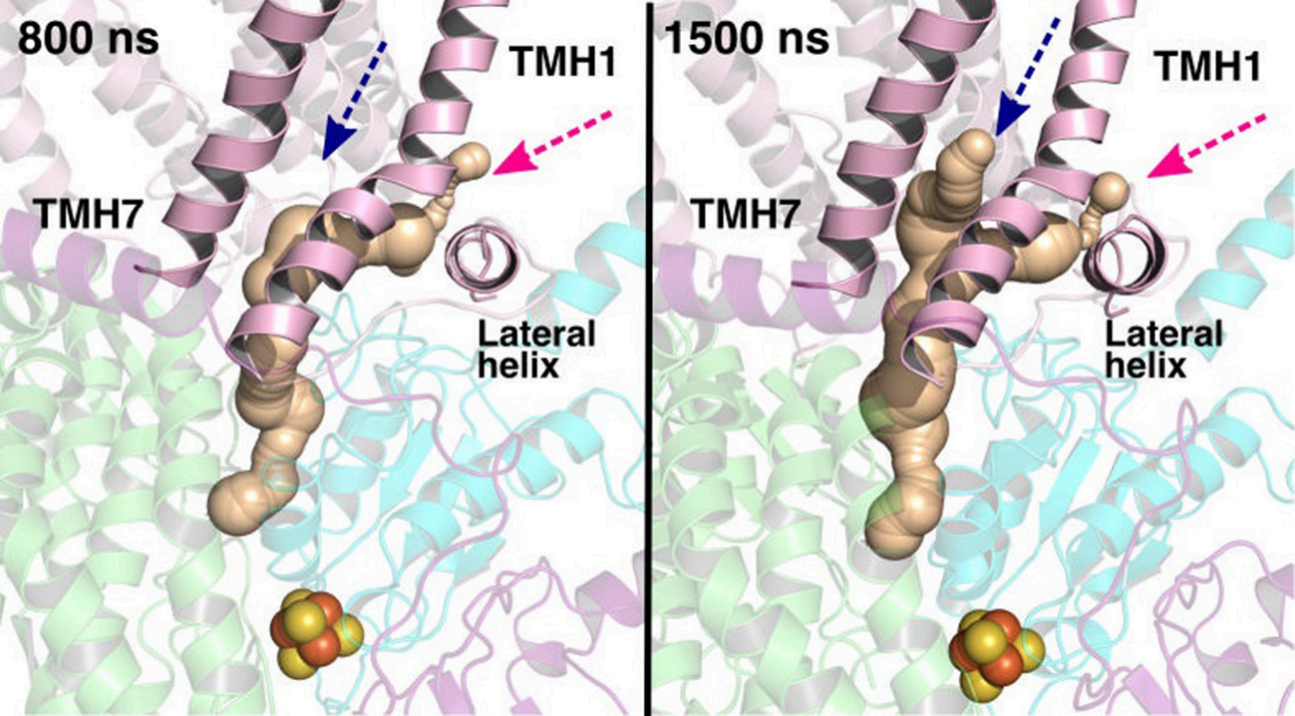
Q binding cavity dynamics shown as simulation snapshots. Both the main (pink arrow) and the alternative Q binding channel (blue arrow) “open” and “close” during simulation without any quinones modeled in the system (system XVI). The alternative cavity is between TMH1 and TMH7 and ”opens” to accommodate Q tail as modeled in system XIII (see Supplementary Figure 4). The probe radius in CAVER was 0.7 Å and the starting point for cavity computation was H38/59 and Y87/108. The main cavity transiently closes during simulation due to the flip of sidechains of hydrophobic amino acid residues (compare to left panel of Figure 12).

The current work advocates the existence of Q binding sites #4 and #5 in addition to well-known binding site near the N2 center [see also (Parey et al., 2018; Warnau et al., 2018)]. The location of Q binding site at the entrance (#5) is in agreement with the preferable Q binding positions in a single and multi-component lipid bilayer, other redox-active membrane proteins (Galassi and Arantes, 2015; Kaurola et al., 2016) as well as with the putative Rotenone binding site (Haapanen and Sharma, 2018). Earlier, mechanistic models have been proposed (Wikstrom et al., 2015; Haapanen and Sharma, 2018; Kaila, 2018; Warnau et al., 2018) in which redox active Q binding sites exist along the Q tunnel. One alternative is to have a trapped Q (or QH_2_) molecule in the Q-tunnel that shuttles between the N2 binding site and site #4, whereas an oxidized Q (yellow in Supplementary Figure 3) at the second site (#5) gets reduced from the shuttling Q (quinol) transiently bound at site #4. Such a scenario has been analyzed earlier based on thermodynamics, molecular modeling and simulations as well as structural arguments (Wikstrom et al., 2015; Haapanen and Sharma, 2017, 2018), and is also supported by the electron density observed (at site #4) in recent structural studies (Parey et al., 2018). Therefore, to shed further light on the dynamics of two Q molecules in the Q cavity [see also (Wikstrom et al., 2015; Haapanen and Sharma, 2018)], we analyzed the structural data and modeled the tail of Q bound near the N2 center through two cavities (Figures 12, 13, see also Zhu et al., 2016). MD runs performed with two Q molecules (at sites #1/5) show that the Q bound near site #1 diffuses, albeit slowly in comparison to single Q in the channel (Figures 2 and Supplementary Figure 4), and approaches the tightly bound Q at site #5 (Supplementary Figure 4B), which may trigger redox-coupled protonation reactions between sites 4 and 5, as also discussed earlier (Haapanen and Sharma, 2018). The data also reveals additional pathways through which a Q tail can pass through (Figures 12, 13), a notion also corroborated by recent chemical biology approaches (Uno et al., 2019) (see above).

### Mechanistic Aspects

Despite a significant number of biochemical, biophysical and structural studies the molecular mechanism of redox-coupled proton pumping by complex I is not known (Hirst and Roessler, 2016; Wirth et al., 2016). Earlier, suggestions have been put forth that involve multiple redox-active sites along the Q tunnel that may be important for proton pumping (Wikstrom et al., 2015; Haapanen and Sharma, 2018). Such a possibility is indeed supported by structural data that show two putative quinone binding sites (Baradaran et al., 2013; Zickermann et al., 2015), and also by computer simulations (Haapanen and Sharma, 2017; Warnau et al., 2018), which revealed two more Q binding sites closer to the Q tunnel opening (see Supplementary Figure 3). Due to the vicinity of N2 cluster to the first site (#1 in Figure 2), it is expected that this is a redox active Q binding site that accepts electrons from N2 FeS cluster in forward mode of complex I catalytic cycle. However, N2 cluster is ca. 25–35 Å from the two other sites (#4 and #5 in Figure 2), and electron transfer from it cannot occur on biological timescales. Therefore, one alternative is that there is a trapped Q molecule in the Q tunnel that shuttles between the two sites (sites 1 and 4) [see also ref. (Wikstrom et al., 2015)]. At site #1, it gets reduced from the N2 cluster (electron input) and at site #4, it reduces another Q (bound at site #5) (electron output), as found in this work and proposed earlier based on thermodynamic, modeling and structural arguments (Wikstrom et al., 2015; Haapanen and Sharma, 2018). Even though no three-dimensional structures are available in PDB that shows two Q molecules bound to complex I, the recently solved cryo EM structure of *Yarrowia lipolytica* complex I (Parey et al., 2018) indeed shows that in addition to a stable decylubiquinone molecule in the Q tunnel near sites 1/2, a second molecule (suggested to be an anionic lipid head group) can bind near site #4; a Q binding region, which we have identified based on simulations (Figure 9; see also Haapanen and Sharma, 2017, 2018). The data supports our viewpoint that two (Q) molecules can bind in the Q-tunnel. Moreover, binding of small Q analogs in the structures of *T. thermophilus* and *Y. lipolytica* complex I also support that small Q molecules (Q_1_) can be trapped in the Q-tunnel (see above). It is not known if the Q cavity is an enclosed one, however, a tight kink at ca. 25 Å from critical Tyr87 (Nqo4) would suggest that cavity may indeed be an excluded one and could trap a Q molecule [see also ref. (Haapanen and Sharma, 2018)].

The redox partner of Q at the Q binding site (#5) could be the trapped Q (QH_2_ or QH^−^ in this case, see Supplementary Figure 3) at site #4. The ≤ 14 Å distance condition for efficient electron transfer from QH_2_ (or QH^−^) at site #4 to an oxidized Q at the site near entrance (#5) is indeed met when a Q molecule is modeled at site #4 [see also ref. (Haapanen and Sharma, 2017)]. The Q molecule, bound at site #5, has its tail fully solvated in lipid bilayer suggesting that its binding may be affected by the lipid composition. We suggest that it would be desirable to steer experimental conditions that may assist in resolving Q (or its analogs) at binding sites 4 and 5, now that the support for sites 1 and 2 exists from structures and simulation data.

The redox and protonation behavior of QH_2_/Q molecules bound at sites 4/5, respectively, are studied by means of quantum chemical simulations. Similar to the Q-binding site near N2 center (site #1), formation of SQ is not coupled to proton transfer, hence, it remains anionic in nature (low p*K*_a_) [see also ref. (Sharma et al., 2015; Gamiz-Hernandez et al., 2017)]. However, transfer of second electron (from Q at site 4) raises the p*K*_a_ much higher resulting in proton transfer from the charged residues of the proton channel in a proton relay kind of mechanism that also involves water molecules (see above). In this way, the Q redox reactions between sites 4/5 bring redox chemistry closer to the central charged axis of the membrane domain of complex I, which may be important in achieving tighter coupling.

A number of different molecular mechanisms have been proposed for complex I. For instance, the proposal by Brandt (2011) involves two Q binding sites, between which the Q molecule shuttles and drives proton pumping. The recent structural and biochemical data (Cabrera-Orefice et al., 2018) support their hypothesis in which concerted movement of loops from ND1, ND3, and PSST subunits couple redox reactions to proton pumping via two-state stabilization change mechanism. However, in this mechanism the source of protons at the Q binding sites and how Q-shuttling couples to antiporter-like subunits at a molecular level remains unknown. In the proposal by Sazanov et al. (Baradaran et al., 2013) that considers single Q binding near N2, reduction of quinone to quinol drives pumping via long-ranged electrostatic and conformational transitions. Similarly, in the single-Q model proposed by Kaila (2018), QH_2_ at site #4 exerts a “push” to the protons in Nqo8 subunit leading to the pumping of protons. We instead suggest that the proton released upon oxidation-coupled deprotonation of QH_2_ (or QH^−^) at site #4 electrostatically “pushes” the protons on membrane-bound subunits via explicit proton transfer in the E channel (Haapanen and Sharma, 2018), a notion that is also considered by Sazanov et al., albeit in their hypothesis E-channel forms part of the fourth proton channel. Our proposal also has mechanistic resemblance to the ideas circulated by Ohnishi et al. (2010) and (Ohnishi and Salerno, 2005) in which EPR-based SQ_Nf_ and SQ_Ns_ species exchange electrons in tight coupling to proton transfer reactions. Currently, it is not possible to determine what kind of conformational transitions would prevent loss in directionality of “pushing” protons in a highly charged and hydrated environment of the Nqo8/ND1 subunit, this will be the subject further studies.

## Conclusions

We have studied here the long timescale dynamics of quinone molecules in complex I using multiscale simulation methods. The observed quinone binding sites in the quinone-chamber agree with the structural data on complex I. Hybrid QM/MM approaches allows us to study the redox-coupled protonation reactions that may occur at Q binding sites closer to the tunnel entrance. The advantage of the proposed Q-shuttle mechanism is that the electron input and output are separated, which may be important in preventing electronic short circuit. Moreover, in this mechanism the Q redox-chemistry occurs much more closely to the membrane domain, making the coupling tighter between the redox reactions and proton pumping.

## Author Contributions

OH and VS designed research. OH, AD, and VS performed research and analysis. OH and VS wrote the manuscript.

### Conflict of Interest Statement

The authors declare that the research was conducted in the absence of any commercial or financial relationships that could be construed as a potential conflict of interest.

## Footnotes

^1^*TURBOMOLE V7.2 2016, A Development of University of Karlsruhe and Forschungszentrum Karlsruhe GmbH, 1989-2007, TURBOMOLE GmbH, Since 2007. Available online at:* *http://www.turbomole.com**. [Online]*.
