# Supplementary material for: Role of Second Quinone Binding Site in Proton Pumping by Respiratory Complex I
Source: Front Chem. 2019 Apr 9;7:221. doi: 10.3389/fchem.2019.00221 (PMC6465577; doi:10.3389/fchem.2019.00221)
Supplement: Supplementary file 1 [file Data_Sheet_1.pdf]

Supplementary Material

1 Supplementary Figures

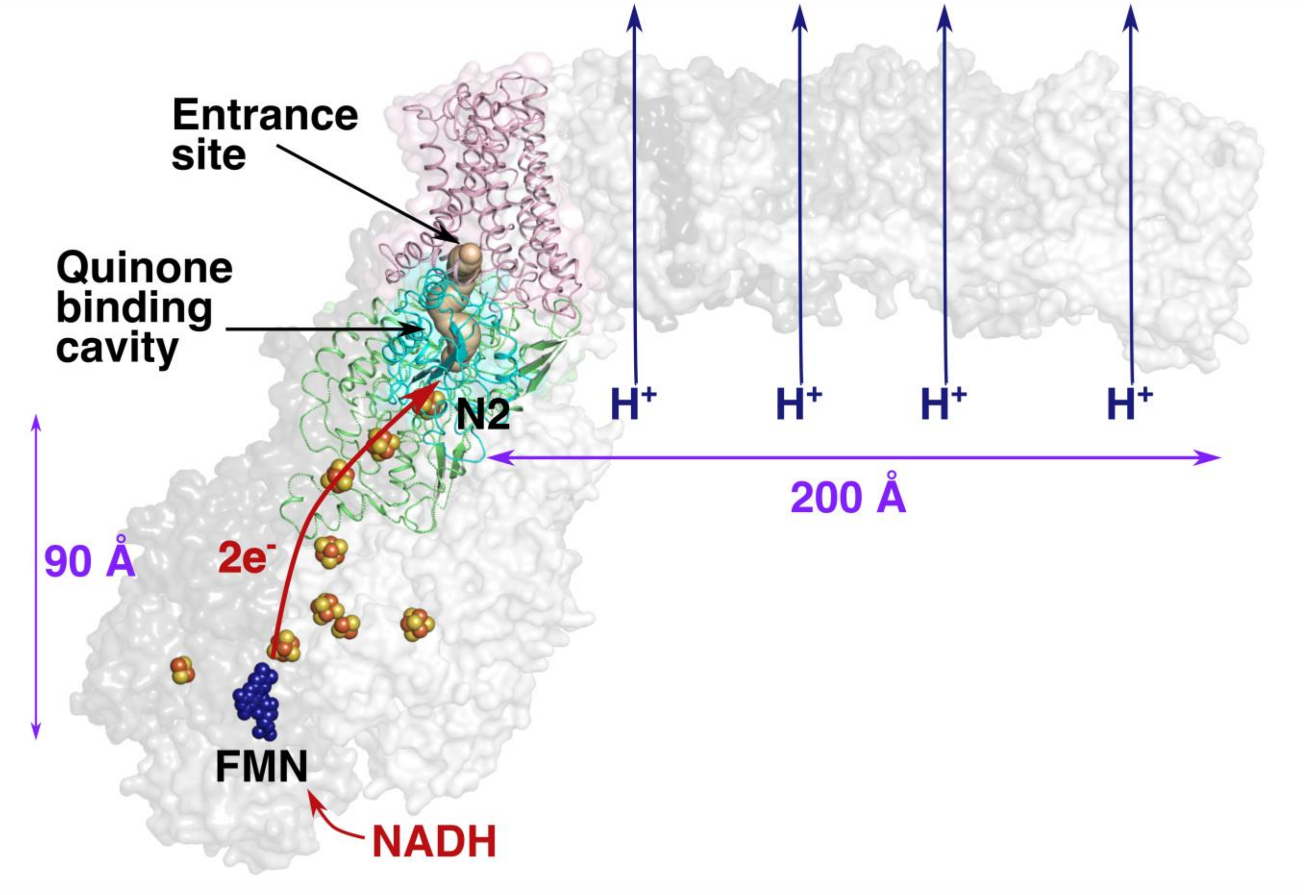

Supplementary Figure 1. The atomic resolution structure of bacterial respiratory complex I from *Thermus thermophilus* (PDB id 4HEA) displayed in surface representation. The electron carriers FeS clusters and FMN are shown, together with the protein subunits Nqo4, Nqo6 and Nqo8 that encapsulate the Q tunnel in green, cyan and pink ribbons, respectively (see also Figure 1). Red and blue arrows depict the electron and proton transfer catalyzed by the enzyme. The electron transfer from NADH/FMN to N2 (ca. 90 Å) is rapid (submilliseconds) and the active site of Q reduction (near N2) is ca. 200 Å away from the furthest proton pumping subunit.

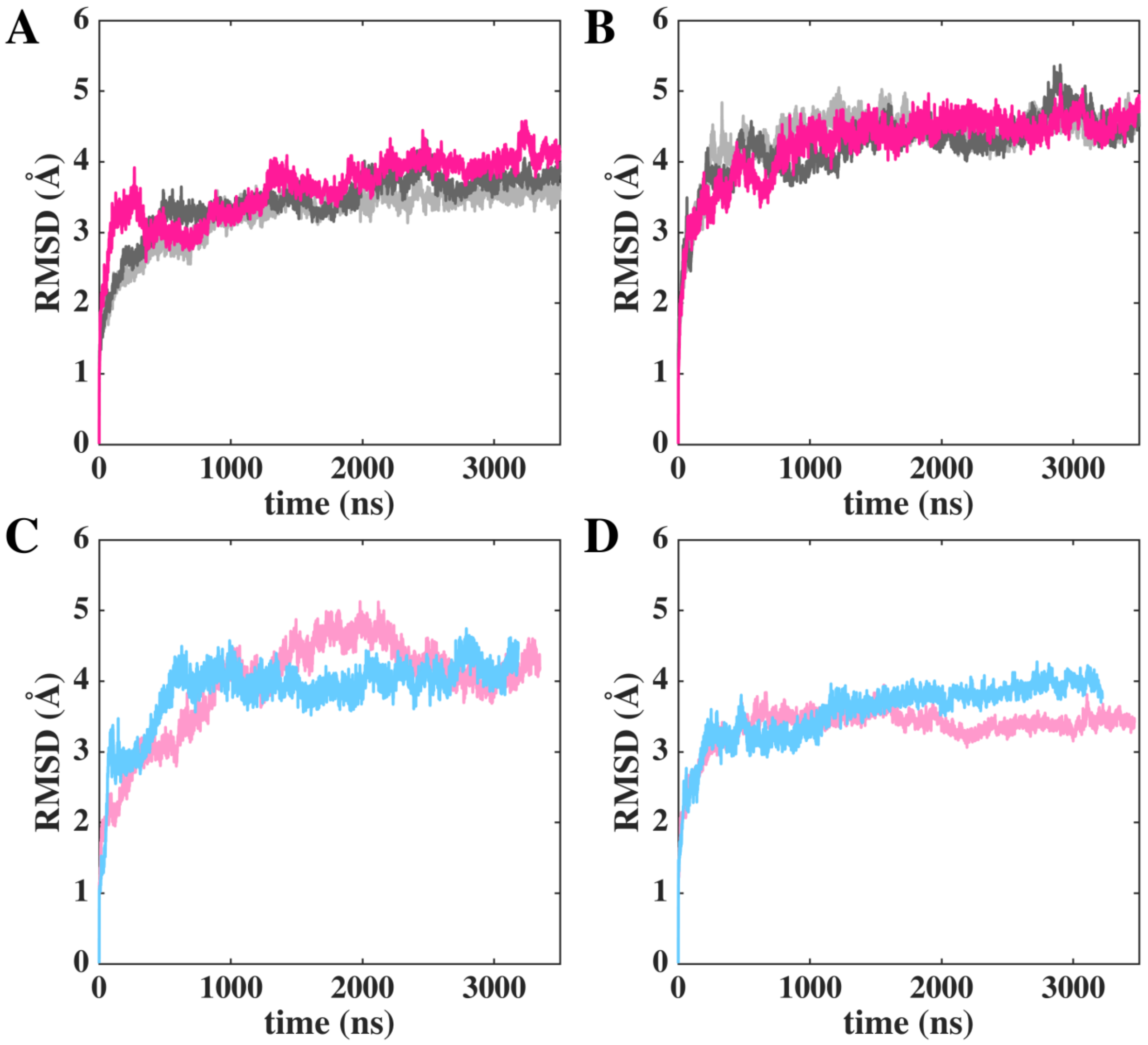

Supplementary Figure 2. Root mean square deviation (RMSD) of setups I (A) and II (B). The three simulation replicas are shown in different colors. RMSD with respect to simulation time is shown in (C) setup III (pink) and setup V (blue) and in (D) setup IV (pink) and setup VI (blue). The simulation trajectories were aligned with respect to the Cα atoms of the energy minimised structure. RMSD was calculated from the aligned trajectories for the subunits that surround the Q cavity: Nqo7/ND3, Nqo8/ND1, Nqo4/49kDa and Nqo6/PSST. Subunits Nqo5/30kDa and Nqo9/TYKY were excluded due to highly dynamic solvent facing loop/termini regions.

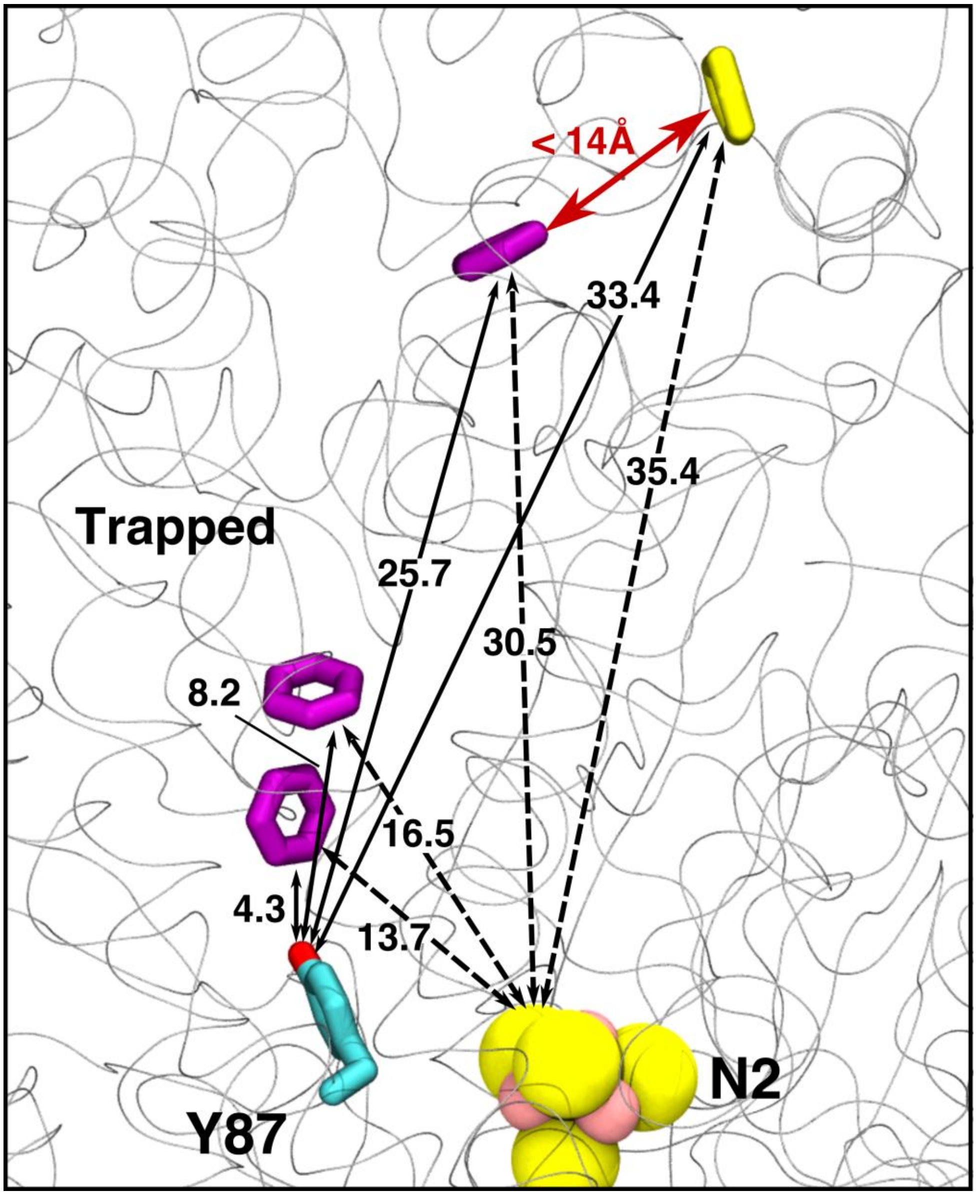

Supplementary Figure 3. Distance of Q head group at various Q binding sites from the N2 FeS cluster and highly conserved Tyr87 (Nqo4) from *T.t.* simulations. The N2-Q distance data and corresponding Tyr87-Q distances are in agreement with the data from umbrella sampling simulations (see main text and Supplementary Figure S11). The Q binding sites 1, 2, 4 and 5, ca. 4, 8, 26 and 34 Å from Y87, respectively, represent the sites 1, 1', 2 and 2'. A high energy barrier is observed for Q diffusion from site 2' to 1, whereas diffusion of oxidized Q between 1, 1' and 2 is nearly barrierless (see ref. Warnau et al., 2018 in main text). Therefore, it is suggested here that a trapped Q molecule shuttles between sites 1, 2 and 4 (purple) and exchanges electrons with another Q (site at the entrance, #5 in Figure 2; Q head group in shown yellow). The reduction of latter Q is coupled to proton uptake from the N side of the membrane, whereas oxidation of QH_2 at site #4 releases protons to conserved amino acid residues close to the "E-channel", as analysed by quantum chemical calculations (see main text).

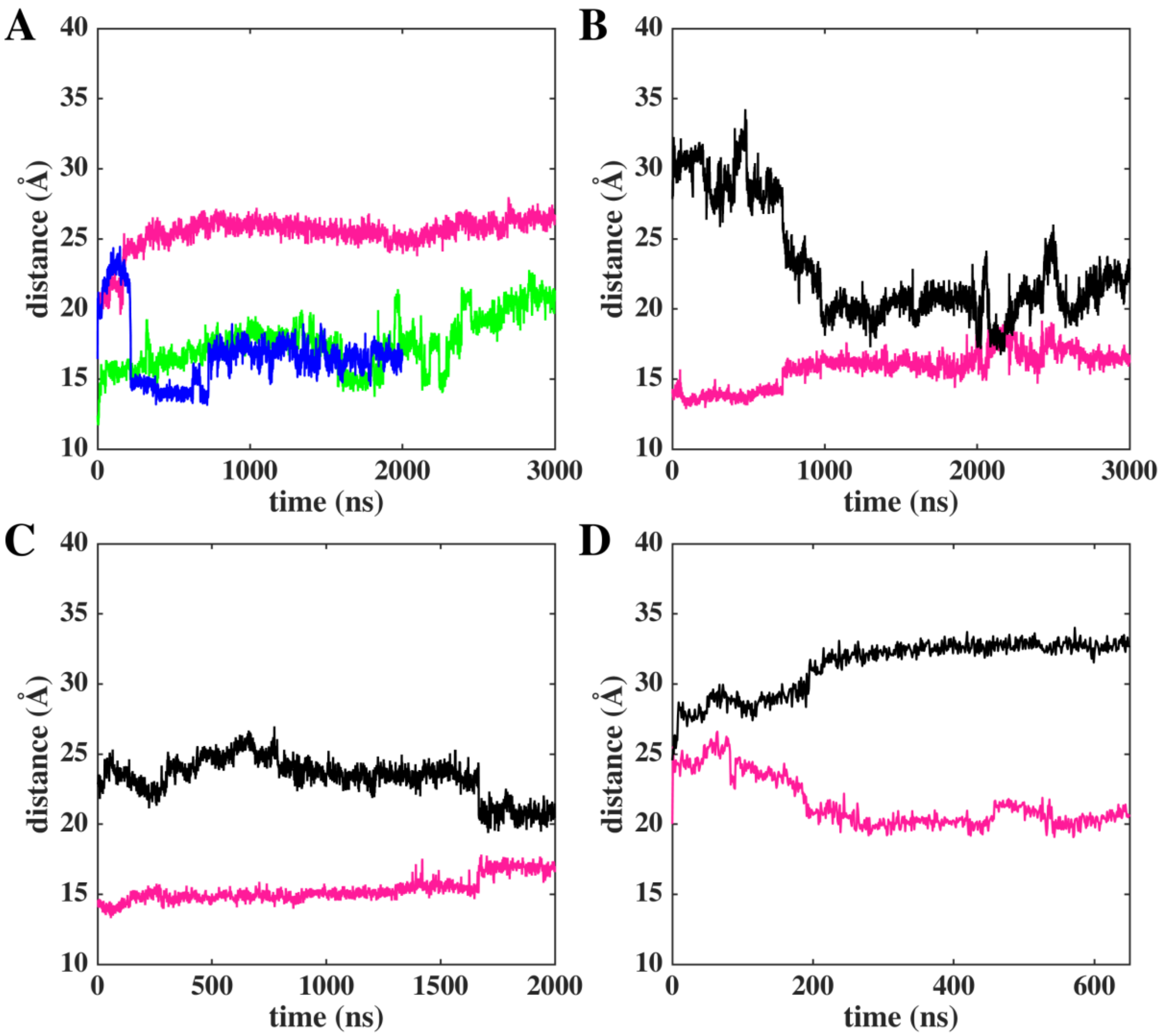

Supplementary Figure 4. (A) Distance of QH_2 headgroup and the N2 iron sulphur cluster in model systems XI (magenta and blue) and XII (green). The QH_2 gets trapped either due to its strong interactions with the acidic residues of 49kD and ND1 subunits or due to unique conformation of β1-β2 (Supplementary Figure 5). To speed-up the dynamics of QH_2, additional simulations were performed (see panel D). (B) Distance of the Q head group from the N2 iron sulphur cluster in system XIII (magenta) and between the head groups of two quinones, one at N2 and another modeled at the E site (#5) (shown in black). The tail of the Q modeled near N2 center was placed through the alternative cavity (Figures 12 and 13, and Supplementary Table 1), and the two Q head groups came within electron transfer distance (black trace; simulation snapshot is available for download at link in Methods section). (C) Same as B for system XIV but with the tail of Q bound at N2 site modeled through the well-known cavity observed in structures, showing slower diffusion of Q near N2 center in contrast to the case in panel B. (D) Distance of QH_2 from the N2 center after initiating QH_2 simulations out of the local traps (see Supplementary Table 1), resulting in rapid diffusion of QH_2 to the site #4 (black trace). In the other case (magenta), it moves to the minimum near site #2. The shown distances are based on the COM (center of mass) of the six carbon atoms of the Q/QH_2 head group ring and the N2 FeS cluster using only Fe and S atoms.

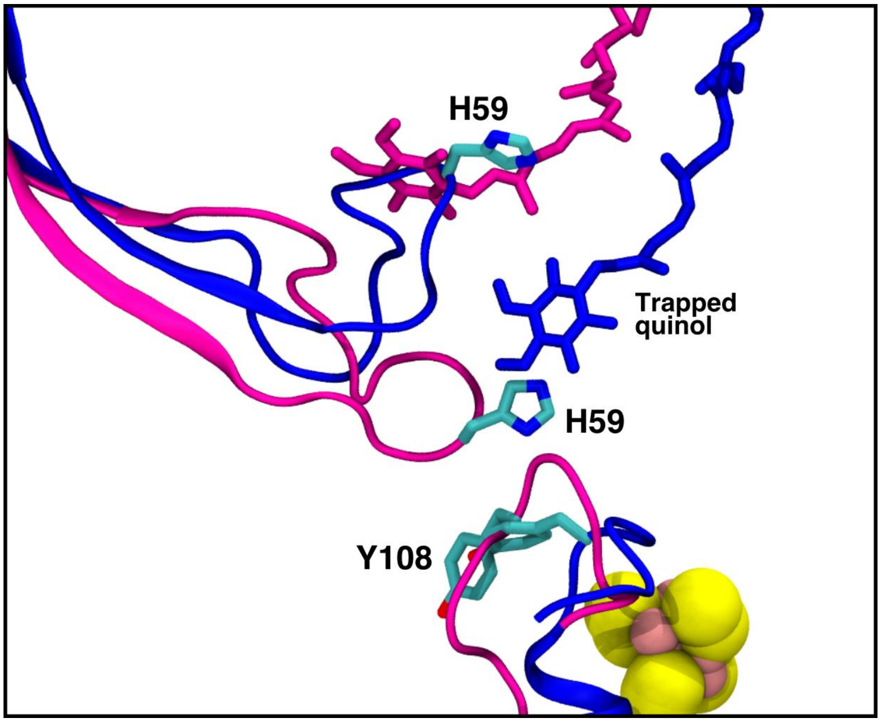

Supplementary Figure 5. Ejection of the QH_2 molecule near the N2 binding site (site #1) is coupled to the dynamics of β1- β2 loop from 49 KDa subunit. When the His59 carrying β1- β2 loop approaches Tyr108 as part of redox-coupled conformational dynamics (see main text), it pushes the QH_2 molecule away from N2. In contrast, QH_2 molecule remains trapped when the β1- β2 loop is in alternative conformation. See Supplementary Figure 4 (panel A, blue and magenta plots, respectively).

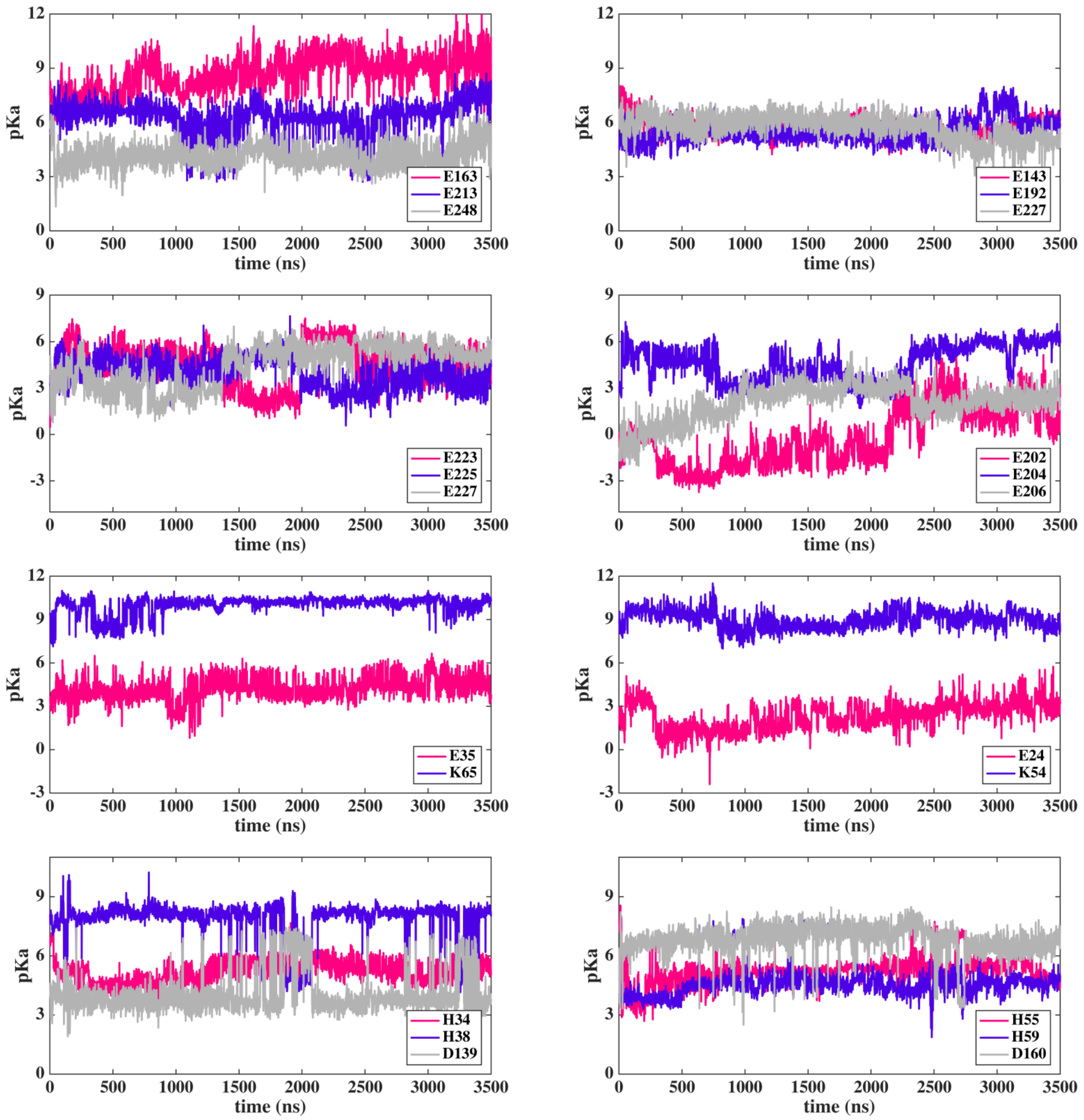

Supplementary Figure 6. pk_a values of selected residues from simulation of *T.t.* and *B.t* enzymes (left and right panels, respectively). The data is calculated from simulation replica shown in magenta color in Figure 2.

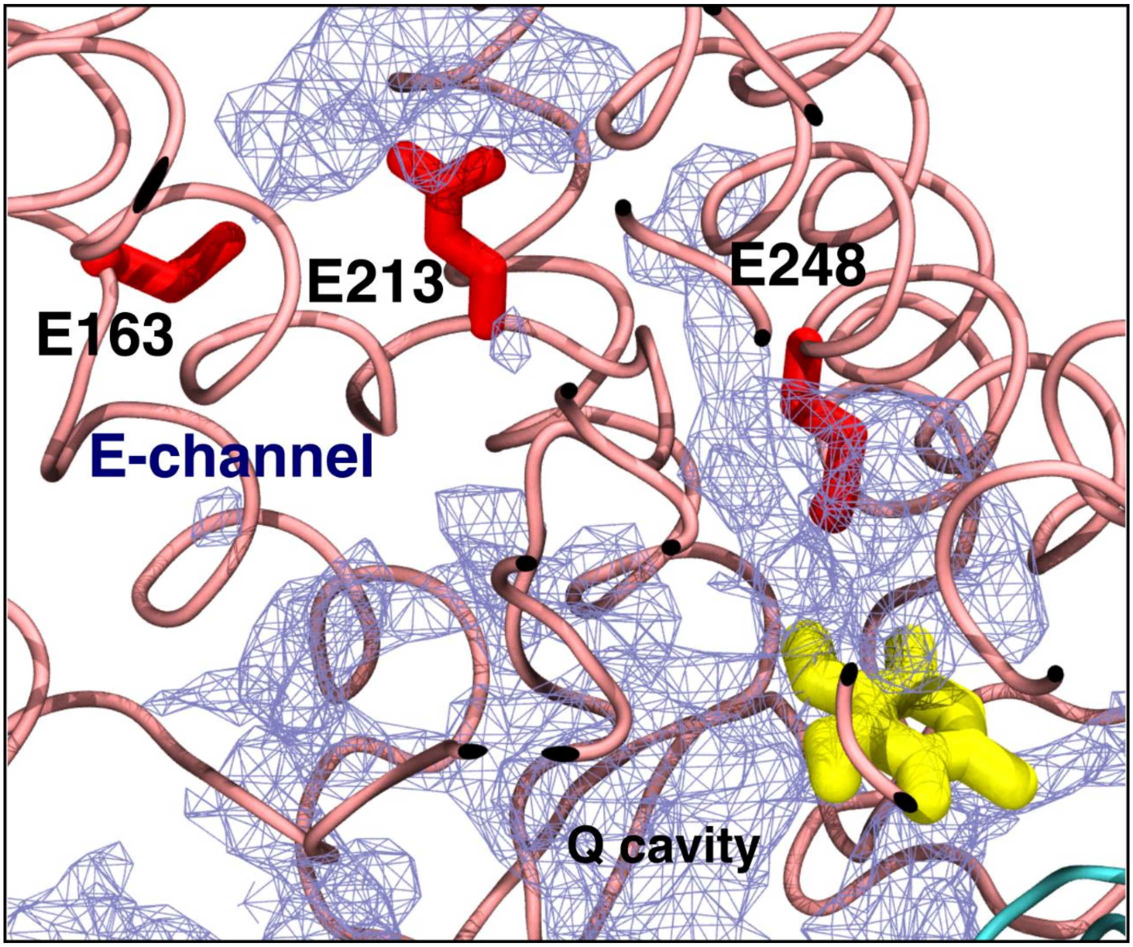

Supplementary Figure 7. Water based connectivity between site #4 and 'E-channel' in *T.t* simulation. Water occupancy is displayed as iceblue mesh using isovalue 0.35. Quinone headgroup is shown in yellow licorice and acidic 'E-channel' residues in red licorice.

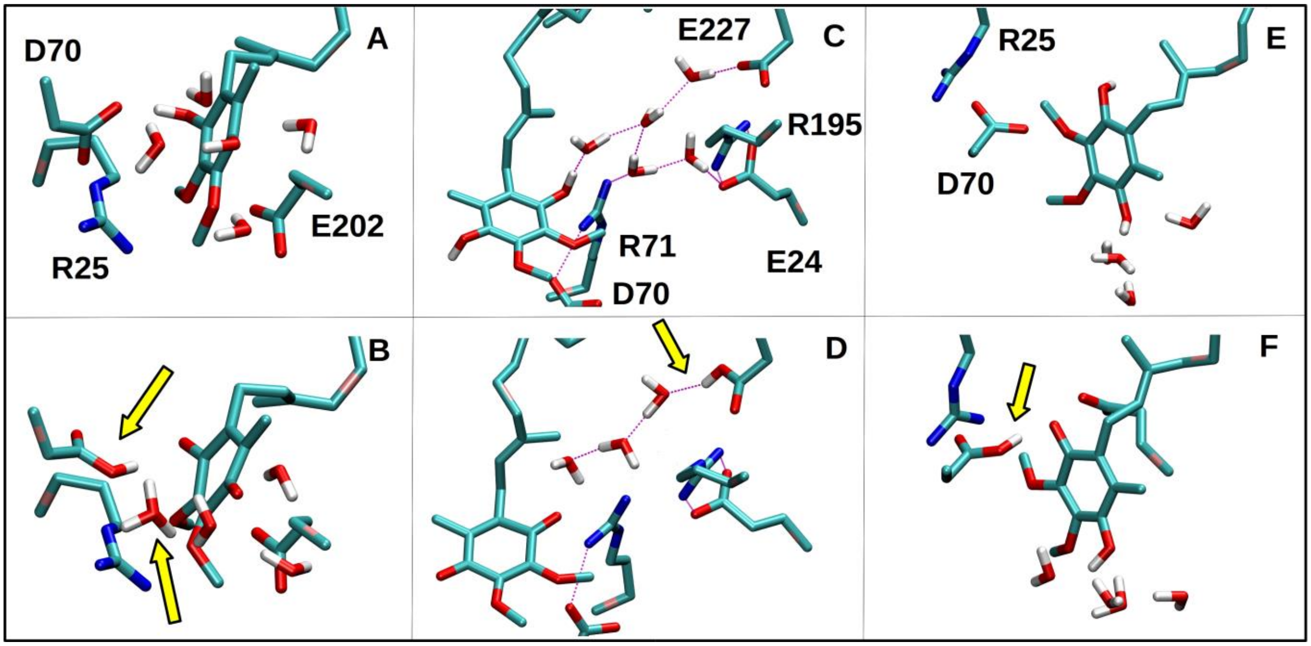

Supplementary Figure 8. QM/MM MD simulations of a QH_2 molecule at site #4 in *B.t.* systems (see also main text and Supplementary Figure 7). Upon two-electron oxidation (panels A to B and C to D) protons from quinol migrate to the nearby conserved residues (D70 in panel B and to E227 in panel D) or to the water cluster (panel B), marked by yellow arrows. In SQ state simulations, both scenarios are observed; formation of a positively charged SQ (E) or a neutral SQ (F). In panel C, QM/MM simulation was performed on a larger model system that also included a key residue (E227) near the "E-channel" that connects to the QH_2 molecule through a water wire and may be important in coupling.

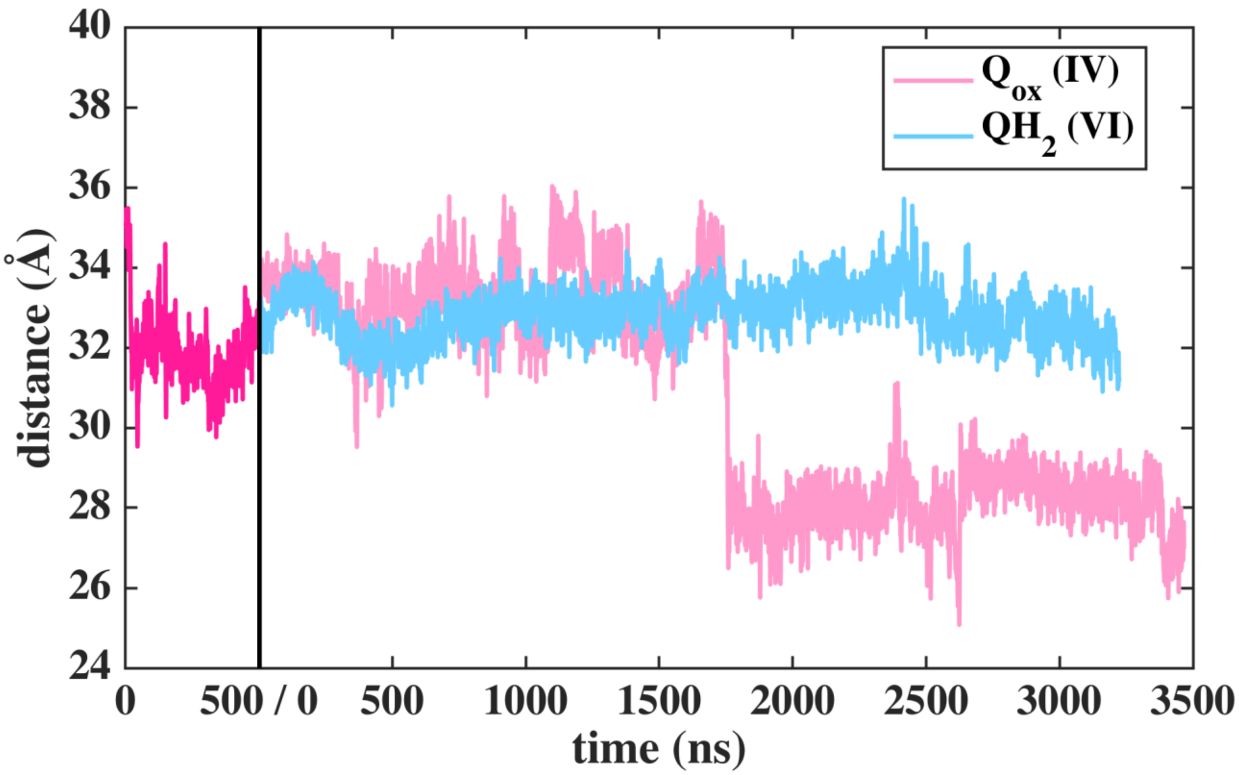

Supplementary Figure 9. Dynamics of two different Q species at the second Q binding site in *B.t.* simulation shown as distance between N2 and Q with respect to simulation time. The dark pink plot corresponds to the last 1500 – 2000 ns of simulation of Q from setup II (see also Figure 2).

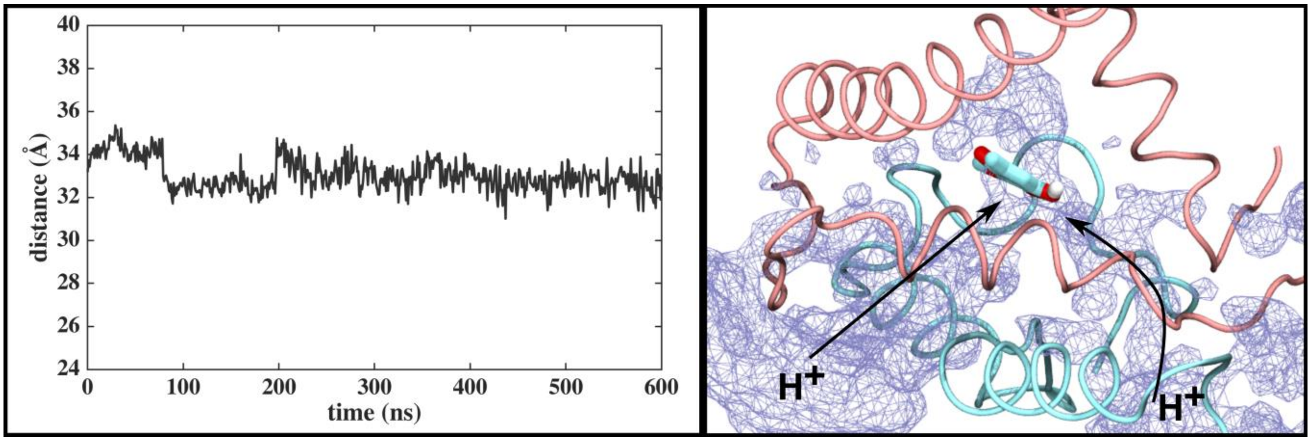

Supplementary Figure 10. MD run of QH^- at the second Q-binding site, where water-protein based paths form between the N side of the membrane and bound Q. Left panel shows the distance between center of mass of Q head group and N2 FeS cluster with respect to simulation time. Right panel shows water occupancy in the region, displayed as ice blue mesh (0.2 isovalue).

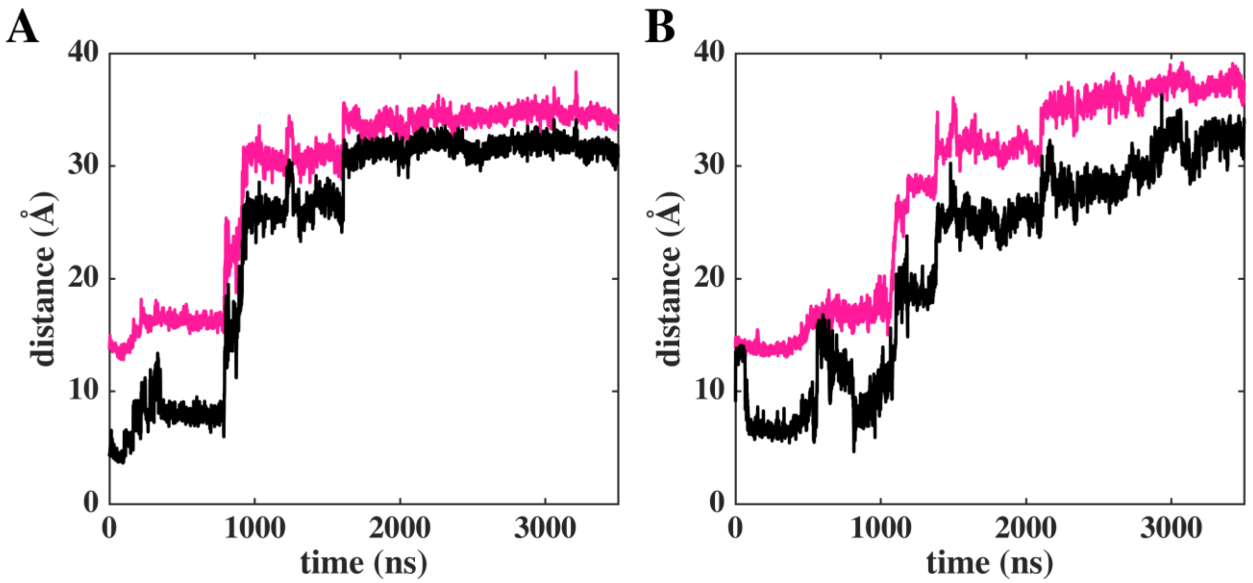

Supplementary Figure 11. Comparison of the distances between the N2 iron sulphur cluster and the quinone headgroup ring (pink traces) and the functionally critical Tyr87/108 and Q head group (black traces) in *T.t.* (A) and *B.t.* (B) simulations. The distance plotted in pink is between the center-of-mass of the six carbon atoms of the Q head group ring and the center-of-mass of N2 FeS cluster (Fe and S atoms) and whereas the distance plotted in black trace is between Tyr (sidechain O atom) and C3 atom of Q head group. The overall profile of pink and black plots matches; however, N2-Q distance plot is more stable due to the missing large side chain fluctuations in Tyr sidechain.

Supplementary Table 1. MD simulation setups and their simulation lengths

Single Q setups				
Setup	Structure (PDB id)	Redox and protonation state of Q	Site at which Q is modeled †	# of replicas x Length (μs)
Q_{10}				
I	4hea	Q_{ox}	N2	3 x 3.5
II	5lc5	Q_{ox}	N2	3 x 3.5
III	4hea	Q_{ox}	E	1 x 3.3
IV	5lc5	Q_{ox}	E	1 x 3.5
V	4hea	QH_2	E	1 x 3.2
VI	5lc5	QH_2	E	1 x 3.2
VII	4hea	QH^-	E	1 x 0.6
VIII	5lc5	QH^-	E	1 x 0.6
IX*	4hea	QH_2	E	1 x 1.5
X*	5lc5	QH_2	E	1 x 1.5
XI	5lc5	QH_2	N2	1 x 3.0 + 1 x 2.0
XII	4hea	QH_2	N2	1 x 3.0 + 1 x 2.0
Double Q_{10} setups				
XIII	4hea	Q/Q ‡	N2/E	1 x 3.0
XIV	4hea	Q/Q	N2/E	1 x 2.0
QH_2 states				
XV	5lc5	QH_2	‡‡	3 x 0.65
Without Q				
XVI	4hea	-	-	1 x 2.5
Q_1				
XVII	4hea	Q	N2	3 x 2.0
XVIII	5lc5	Q	N2	3 x 2.0

† N2 or E refers to the site where a Q molecule was initially placed that is the N2 binding site (#1 in Figure 2) or the second Q binding site at the entrance (#5 in Figure 2), respectively.

*QH_2 simulation by taking a snapshot from Q_{ox} MD runs of setup I and II. ‡ The tail of Q molecule at the N2 binding site (site #1 in Figure 2) is modeled through the alternative cavity observed in complex I structures and simulations (see Figures. 12 and 13).

‡‡ QH_2 was modeled between sites #2 and #3 by taking snapshots from Q_{ox} simulations (setup II) and two 0.65 μs simulations were performed. An additional 0.65 μs simulation was performed by modeling two acidic residues (Asp160 of 49 kDa and Glu204 of ND1) protonated, which have been suggested to be important for protonation of proton-holes created upon QH_2 formation at site # 1. The

data in panel D of Supplementary Figure 4 shows diffusion of QH_2 molecule in the tunnel to sites 4/5, similar to Q dynamics.

Supplementary Table 2. QM/MM MD simulation setups and their simulation lengths

Setup* (site where Q is modeled)	Redox state of quinone	Protonation state of specific residues (subunit)	Simulation length (~ps) †
B1 (site # 5)	Q	-	1.6 + 0.8
	SQ		1.0 + 0.6
	Q^{2-}		1.6 + 0.8
B2 (site # 5)	Q	Glu59 (ND1) protonated	0.7
	SQ		0.3
	Q^{2-}		0.6
B3 (site # 5)	Q	Asp51 (ND1) protonated	1.4
	SQ		0.8
	Q^{2-}		1.5
B4 (site # 4)	QH_2	-	1.5
	SQ		1.0
	Q		1.5
B5 (site # 4)	QH_2	-	1.5
	SQ		0.5
	Q		1.1
B6 (site # 4)	QH_2	-	1.8
	SQ		0.7
	Q		1.6
T1 (site # 5)	Q	-	2.1 + 2.0
	SQ		1.3 + 1.3
	Q^{2-}		2.2 + 2.1
T2 (site # 5)	Q	Asp76 (Nqo6) protonated	1.8
	SQ		1.0
	Q^{2-}		1.4
T3 (site # 5)	Q	Asp59 (Nqo6) protonated	0.9 + 0.8
	SQ		0.4 + 0.4
	Q^{2-}		0.5 + 0.3

* B1-B6 and T1-T3 setups correspond to *B.t.* and *T.t.* enzymes, respectively.

† Additional simulation replicas are separated by a "+" sign

Video S1. Coupling between Q diffusion and protein dynamics from simulation setup I. Q diffuses from the N2 binding site (#1 in Figure 2) to a site ca. 35 Å from N2 (#5 in Figure 2) next to the lateral helix (residues 55-69 from Nqo8 shown in purple ribbons). Amino acid residues 210-250 in light pink, 17-59 in green and 60-85 in cyan are from subunits Nqo8, Nqo4 and Nqo6, respectively. Quinone is shown in yellow licorice and N2 in yellow-pink vdW spheres. The movement of Q is tightly coupled to backbone conformational dynamics of Nqo6 subunit, which shows an RMSF of Cα atoms (residues 60-85) ~ 2.1 Å in contrast to ~ 1.4 Å, when Q stays. RMSF of all Cα atoms is summed and divided by number of atoms.

Video S2. Coupling between Q diffusion and protein dynamics from simulation setup II. Q diffuses from the N2 binding site (#1 in Figure 2) to a site ca. 35 Å from N2 (#5 in Figure 2) next to the lateral helix (residues 45-59 from ND1 shown in purple ribbons). Amino acid residues 190-230 in light pink, 37-79 in green and 74-94 in cyan are from subunits ND1, 49kD and PSST, respectively. Quinone is shown in yellow licorice and N2 in yellow-pink vdW spheres. The movement of Q is tightly coupled to backbone conformational dynamics of PSST subunit, which shows an RMSF of Cα atoms (residues 74-94) ~ 1.6 Å in contrast to ~ 0.9 Å, when Q stays. RMSF of all Cα atoms is summed and divided by number of atoms.

Video S3. Semiquinone formed after one-electron reduction of Q at the second Q-binding site remains anionic in nature (see also Figure 10), in contrast to the rapid protonation of quinol (Q^{2-}) species (see video S4). Amino acids, quinone and water molecules are shown in CPK representation. Backbones of Nqo6 (cyan) and Nqo8 (light pink) are shown in thin transparent ribbons. A cutoff of 1.6 Å is chosen to display the proton dissociation and association during its transfer along water molecules and residues in Videos S3-S5.

Video S4. Doubly reduced quinol (Q^{2-}) abstracts proton from the conserved K65 already during minimization and is stabilized as QH^-. Amino acids, quinone and water molecules are shown in CPK representation. Backbones of Nqo6 (cyan) and Nqo8 (light pink) are shown in thin transparent ribbons.

Video S5. Reprotonation of K65 from D76 that faces the aqueous phase at the N side of the membrane, suggesting Q-reduction coupled proton uptake at the second Q binding site. Amino acids, quinone and water molecules are shown in CPK representation. Backbones of Nqo6 (cyan) and Nqo8 (light pink) are shown in thin transparent ribbons.
